# Parasporin-2-Derived Peptide Fragments: Characterization and Synergistic Anticancer Activity with Sacha Inchi and Curcumin

**DOI:** 10.3390/cancers18030451

**Published:** 2026-01-30

**Authors:** Natalia Ardila, Fanny Guzmán, Miguel O. Suárez-Barrera, Jenniffer Cruz

**Affiliations:** 1 Instituto de Investigación Masira, Facultad de Ciencias Médicas y de la Salud, Universidad de Santander, Bucaramanga 680001, Colombia; biomol.investigacion@udes.edu.co (N.A.); miguel.suarez@udes.edu.co (M.O.S.-B.); 2 Curauma Biotechnology Center, Pontificia Universidad Católica de Valparaíso, Valparaíso 2362807, Chile

**Keywords:** parasporins, anticancer peptide, peptide synthesis, natural compounds, synergism

## Abstract

Background/Objectives: Parasporin PS2Aa1, recently designated as Mpp46Aa1, is recognized for its selective anticancer activity against various human cell lines. In this study, specific regions of the native protein were fragmented, and targeted amino acid substitutions were introduced to improve cytotoxic selectivity and potency. Methods: The modified fragments were evaluated individually and in combination with curcumin, a polyphenol with well-documented anticancer properties, and Sacha inchi-derived matrices, known for their antioxidant and antiproliferative activities. Results: Experimental results demonstrated that the substituted variant designated T104L-G108W exhibited superior anticancer activity compared to the native peptide P102-K11. Synergism assays revealed that curcumin-bioconjugated peptides were more effective against the tested cell lines, whereas combinations with Sacha inchi reduced cytotoxicity, suggesting possible interference in the mechanisms of action. Functional assays, including caspase 3/7 and 9 activation, Annexin V-Cy3 staining, and cell viability analysis with 6-CFDA, confirmed increased sensitivity in SiHa and HeLa cell lines, particularly for peptide T104L-G108W. Conclusions: Collectively, these findings support the effectiveness of a substitution-based strategy in improving parasporin fragments and underscore the therapeutic potential of peptide T104L-G108W as a novel anticancer candidate. Furthermore, this study provides preliminary evidence that natural biomolecules can be optimized through targeted modifications and rational combinations, establishing a framework for the development of sustainable and selective therapeutic approaches in cancer treatment.

## Simple Summary

Cancer remains one of the leading causes of mortality worldwide, underscoring the urgent need for innovative and effective therapeutic strategies. Parasporins, a group of proteins produced by *Bacillus thuringiensis*, have gained considerable attention due to their selective cytotoxicity toward cancer cells while sparing healthy tissues. Curcumin, a polyphenolic compound from turmeric, and Sacha inchi, an Amazonian seed rich in polyunsaturated fatty acids and bioactive molecules, also exhibit well-documented antioxidant, anti-inflammatory, and antiproliferative properties. The objective of this study was to characterize peptide fragments derived from Parasporin-2 and evaluate their anticancer activity, both individually and in combination with curcumin and Sacha inchi, to determine potential synergistic effects. To achieve this, we conducted in vitro cytotoxicity and antiproliferative assays on human cancer cell lines using peptide fragments, curcumin, and Sacha inchi, alone and in combination, to assess their integrated biological activity and quantify synergism. This is the first study to explore the combined effects of Parasporin-2 peptides with curcumin or Sacha inchi, offering new insights into natural and peptide-based synergistic platforms for potential anticancer therapy.

## 1. Introduction

Cancer remains one of the leading causes of mortality worldwide and represents a persistent challenge due to its biological heterogeneity, recurrence, and resistance to conventional therapies [1]. Globally, according to GLOBOCAN 2022 [2], 19.3 million incidents and millions of deaths from cancer were reported. Of these cases, breast cancer remains the most frequently diagnosed malignancy, with approximately 2.3 million new cases (11.7% of all cancers), and is the leading cause of cancer mortality in women, with 665,000 deaths annually. Colorectal cancer is the third most common cancer globally, with 1.9 million new cases (9.7%), and represents the second leading cause of cancer death overall, with nearly 904,000 deaths annually, underscoring its considerable case fatality. In contrast, cervical cancer, although less prevalent, with 660,000 new cases, imposes a disproportionately high mortality burden, causing 348,000 deaths annually. It is the fourth most common cancer among women but remains the leading cause of cancer-related mortality in women in 36 countries, predominantly low- and middle-income nations [2].

The search for novel anticancer therapies has intensified, highlighting the potential of *Bacillus thuringiensis* (*Bt*) parasporins. A prominent candidate, PS2Aa1 (Mpp46Aa1), has garnered significant interest for its selective cytotoxicity against cancer cells. This protein is a protoxin, activated by proteinase K cleavage, which belongs to the β-pore-forming toxin family. Its anticancer mechanism is multifaceted: it binds to specific membrane receptors, oligomerizes to form pores, and induces mitochondrial depolarization. This cascade leads to phosphatidylserine externalization, activation of caspases (including caspase-9), and ultimately triggers the intrinsic pathway of apoptosis [3,4,5]. Following this potential, the Molecular Biology and Biotechnology (BIOMOL) research group at the University of Santander (UDES) has previously investigated PS2Aa1, identifying promising mutants such as 3–35 (G257V) and N65 (N92D, K175R, S218G) [6,7,8].

In other contexts, the research of natural bioactive compounds has gained relevance due to their multiple mechanisms of action and comparatively lower toxicity compared to synthetic drugs. Curcumin, a polyphenol from Curcuma longa, exhibits antioxidant, anti-inflammatory, and antitumor activities by modulating signaling pathways associated with proliferation, apoptosis, and oxidative stress [9]. Despite its therapeutic potential, curcumin’s low solubility, rapid metabolism, and limited bioavailability remain major obstacles to its clinical application [10]. In parallel, Sacha inchi (*Plukenetia volubilis*), an Amazonian plant of growing scientific interest, is recognized as a sustainable source of polyunsaturated fatty acids, proteins, and bioactive peptides. Sacha inchi extracts and protein hydrolysates have demonstrated antioxidant, anti-inflammatory, and antiproliferative properties, with beneficial effects on metabolic parameters and tumor cell viability [11].

Furthermore, peptide-based drug conjugates (PDCs) have been identified as candidates to be used in cancer drugs, given their potency to act by targeting tumor-expressed receptors that are overexpressed for cancer therapies with reduced systemic toxicity. Several studies have shown that PDCs can be used as a targeted anticancer drug against cancer tissue, using cytotoxic compounds with peptide carriers.

One well-documented investigation by Lelle et al. employed an octreotide-targeted doxorubicin–peptide conjugate to induce somatostatin receptor activation in cancer cells. An effective intracellular delivery of the cytotoxic drug, through receptor-mediated endocytosis, was accomplished as a result of this conjugate, which underlines the necessity of receptor specificity for increasing drug use in tumors [12]. In a similar vein, the peptide–drug conjugate TH1902 that acts on the sortilin receptor elicited inhibition of tumor growth in models of ovarian and endometrial cancer, further validating the importance of receptor-targeted regimes for improving treatments with regard to clinical outcomes in several malignancies [13].

Developments of PDCs were further attributed to the design of the linker systems linking peptides to cytotoxic drugs. According to Zhu et al., these linkers are instrumental in the stability and circulation of PDCs in the blood, while permitting the controlled release of drugs to the target [14]. Further investigation of linker chemistry has supported the synthesis of new kinetics and forms of conjugates (e.g., keratin-1-targeting doxorubicin-based ones) in addition to increasing the uptake and efficacy in triple-negative breast cancer models [15].

Apart from traditional chemotherapeutic agents, a myriad of studies have shown that PDCs can be utilized to deliver bioactive agents, like siRNA and small-molecule inhibitors [16]. For instance, peptide sequences engineered for the presence of tumor receptors facilitated their application in a therapeutic setting, promoting greater cellular uptake and overcoming drug resistance mechanisms frequently observed in solid tumors [14]. In addition, the advancement of conjugation strategies and peptide design permitted selective inhibition of tumor growth and metastasis and attenuated side effects of systemic therapies [17]. The exploration and optimization of peptide–drug conjugates is an evolution in targeted cancer therapies. Such methods leverage the specific properties of peptides, resulting in a targeted treatment against neoplasms and a superior therapeutic index, and, ultimately, better clinical oncology results.

However, based on the available literature, this appears to be the first report exploring the combined use of Parasporin-derived peptides bioconjugated with curcumin together with components derived from Sacha inchi in the context of cancer therapy. Addressing this gap is of particular relevance, as this approach could provide a synergistic strategy integrating three complementary actions: the intrinsic bioactivity of curcumin, the improved solubility, bioavailability, and selectivity conferred by peptide conjugation, and the nutritional and functional support of Sacha inchi lipids and proteins as natural carriers and adjuvants. Based on this rationale, the present study focuses on the design and characterization of curcumin–peptide bioconjugates incorporated into Sacha inchi lipid–protein matrices, with the aim of evaluating their potential as innovative therapeutic systems against cancer cell lines. We hypothesize that short PS2Aa1-derived peptide fragments, although structurally incomplete, may retain partial membrane interaction or bioactive potential, and that their anticancer activity could be enhanced by co-treatment with natural bioactives (curcumin or Sacha inchi) through complementary apoptotic or redox-mediated pathways.

## 2. Materials and Methods

### 2.1. Materials and Reagents

The synthesis was performed using Fmoc-Rink Amide AM resin (0.55 mmol/g) with standard Fmoc-protected amino acids activated by HBTU, HCTU, DIPEA, or OxymaPure^®^. Deprotection was carried out using 4-methylpiperidine (4MP), and cleavage was performed with trifluoroacetic acid (TFA) in the presence of trisopropylsilane (TIS) and 2,2′-(ethylenedioxy) diethanethiol (DOT) as scavengers. Solvents used included N,N-dimethylformamide (DMF), isopropanol (IPA), dichloromethane (DCM), and synthetic-grade diethyl ether, as well as HPLC-grade methanol, acetonitrile, and ethanol.

All chemical reagents and curcumin were purchased from Merck KGaA (Darmstadt, Germany), except for the resin, amino acids, and coupling activators, which were obtained from Iris Biotech GmbH (Marktredwitz, Germany). 5-Fluorouracil (5-FU), fetal bovine serum (FBS), Alamar Blue HS (high sensitivity), Annexin V-Cy3, Triton X-100, and Dulbecco’s Modified Eagle’s Medium (DMEM) were purchased from Sigma-Aldrich (St. Louis, MO, USA). L-glutamine, penicillin–streptomycin, and Trypsin–EDTA (1×) were obtained from Invitrogen/Gibco (Waltham, MA, USA). Caspase-Glo^®^ 3/7 and Caspase-Glo^®^ 9 assay kits were acquired from Promega (Madison, WI, USA).

### 2.2. Designs of Peptide Fragments Derived from PS2Aa1

An in silico study was conducted to design and evaluate peptides derived from the parasporin protein PS2Aa1, previously characterized for its anticancer potential by the Molecular Biology and Biotechnology research group at the University of Santander [6,7,8]. Using PS2Aa1 and its mutant variants as templates, peptides were designed from distinct regions of the protein. These peptides were analyzed using a set of bioinformatics tools to predict anticancer activity (AntiCP 2.0), toxicity (ToxinPred 3.0), and physicochemical properties (ProtParam: https://web.expasy.org/protparam/ accessed on 5 January 2026 and, Innovagen: https://pepcalc.com/ accessed on 5 January 2026) [18,19,20]. Considering the sequences of the four previously selected peptides, a computational algorithm was developed in Python 3.5 to identify potential analogs containing two mutations. Subsequently, the Python package AntiCP2 was used to select those peptides with a predicted anticancer activity probability higher than 0.7, and only those with a predicted toxicity probability lower than 0.1 were retained [21].

Three-dimensional structures were modeled (PEP-FOLD 3.5) [22], validated (Swiss-Model: SWISS-MODEL, MolProbity: MolProbity bio.tools) [23], and visualized (UCSF Chimera 1.19) [24]. The scoring of peptides generated by the Swiss model evaluation is provided in the Supplementary Material. The integrated data established a comparative profile of the candidate sequences, prioritizing those with the most promising combination of structural stability, low toxicity, and high predicted anticancer activity potential for further investigation.

### 2.3. Peptide Synthesis

All peptide fragments derived from PS2Aa1 and its analogs were synthesized using Fmoc-based solid-phase peptide synthesis (SPPS) according to the principle of Circular Economy [25].

The peptide synthesis procedure in “tea bag” style bags begins with swelling the resin in polyethylene bottles using DCM and DMF for 30 min each, while verifying seal integrity and label legibility. Subsequently, an initial deprotection is carried out through two cycles with 4MP solution for 10 min at room temperature, followed by washes with DMF, IPA, and bromophenol blue [25].

Each coupling cycle starts with deprotection under a reagent recycling scheme, followed by a single coupling with an amino acid, HBTU/OxymaPure, and DIPEA (10 min at 45 °C and 3 h at 25 °C), and a double coupling with HCTU and DIPEA for 1 h, reusing the washing solutions. Progress is evaluated through a colorimetric test with bromophenol blue: blue resin indicates Fmoc removal; blue/green indicates incomplete coupling; and yellow/orange indicates complete coupling. If blue or green tones persist, coupling is repeated with another activator [25].

Approved bags are washed with DMF, and the cycle is repeated until the sequence is complete. Once synthesis is finished, a final deprotection and cleavage is performed using TFA:TIS:H_2_O (95:2.5:2.5) or with additional DOT for peptides containing Cys, Met, or Trp, incubating for 3 h at room temperature with agitation. The obtained peptide is precipitated with cold diethyl ether, centrifuged, and washed four times; the pellets are dried, dissolved in milliQ water, fractions are combined, frozen, and, finally, lyophilized for 48 h [25].

### 2.4. Peptide Purification and Structural Characterization

Peptide purification was performed by reverse-phase high-performance liquid chromatography (RP-HPLC) (Jasco Corporation, Tokyo, Japan) employing a Vydac C18 preparative column. The mobile system consisted of solvent A (water with 0.1% trifluoroacetic acid, TFA) and solvent B (acetonitrile with 0.1% TFA, *v*/*v*). Peptide elution was achieved under a linear gradient from 5% to 70% of solvent B over 30 min, at a flow rate of 1.0 mL/min, with UV detection monitored at 220 nm. The molecular weight of the purified fractions was subsequently verified by electrospray ionization mass spectrometry (ESI-MS) [25].

Secondary structural features were assessed by circular dichroism (CD) spectroscopy at 25 °C, using a 1 mm path length quartz cuvette. Spectra were collected between 190 and 260 nm with a J-815 CD spectropolarimeter (Jasco Corporation, Japan), employing peptide solutions at 0.2 mM in 50 mM sodium phosphate buffer (pH 7.4) supplemented with 30% (*v*/*v*) 2,2,2-trifluoroethanol (TFE). Each CD spectrum represented the average of three consecutive scans, acquired at a scanning speed of 20 nm/min and a bandwidth of 1 nm. All experiments were independently repeated four times, and the results were reported as averaged values [25].

### 2.5. Conjugated Peptide Synthesis

All peptides derived from the PS2Aa1 sequence were bioconjugated with curcumin through Fmoc-based solid-phase peptide synthesis (SPPS), following the Circular Economy approach [25]. Bioconjugation was achieved by forming a covalent amide bond between the N-terminal amino group of each peptide and a carboxyl group previously introduced into curcumin via the functionalization of one of its phenolic moieties [25].

Reaction “tea bag” synthesis pouches were used. The process began by swelling the resin in DCM and DMF. The synthesis itself started with an initial Fmoc deprotection step, followed by optimized coupling cycles: first, a single coupling using HBTU/OxymaPure, and, subsequently, a double coupling with HCTU, reusing reagent solutions to minimize waste. The progress of each step was monitored by a bromophenol blue colorimetric test. Upon completing the peptide sequence, the modified curcumin was coupled through a triple-coupling strategy to ensure full conjugation [25].

After synthesis, peptides were deprotected and cleaved from the resin using a TFA/TIS/H_2_O (95:2.5:2.5) cocktail, or with additional DOT for sequences containing Cys, Met, or Trp, incubated for 3 h at room temperature. The crude peptide was precipitated in cold diethyl ether, thoroughly washed, and dried. It was then redissolved in Milli-Q water, pooled, and lyophilized for 48 h [25].

### 2.6. Sacha Inchi/Peptide Mixture

The peptides and Sacha inchi oil were formulated in a 1:1 (*w*/*w*) ratio at a total concentration ranging from 5 to 200 µg/mL. For each concentration, the peptide stock solution (1 mg/mL in DMEM with 10% FBS) was mixed with the lecithin-stabilized oil emulsion (10 mg/mL) and then diluted in DMEM supplemented with 10% FBS.

### 2.7. Hemolytic Activity Assay

The hemolytic activity peptides were determined by measuring the hemolysis induced by them in sheep erythrocytes [6]. 1% Triton X-100 was used as a positive control. The percentage of hemolysis was calculated using the following equation.

% Hemolytic Activity = [(Amx − Anc)/(Acp − Anc)] × 100

where Amx is the absorbance of the sample, Anc is the absorbance of erythrocytes in the absence of lysis, and Acp is the absorbance of erythrocytes after complete lysis.

### 2.8. Cell Culture Conditions

The colorectal cells (SW480, SW620, and NCM460), human breast cancer cell line (MCF-7), epithelial cervix adenocarcinoma cell line (HeLa), and squamous carcinoma cells (SiHa) used in this research were obtained from the Colombian scientific NanoBioCancer Program. All cell lines have grown in a 75 cm^2^ flask and maintained 10 mL Dulbecco’s Modified Eagle’s Medium-High Glucose DMEM, with 10% fetal bovine serum (FBS), 1% 2 mM L-glutamine, 1% MEM of non-essential amino acids, and 1% penicillin–streptomycin 10,000 U/mL–10,000/mL. Cells were cultured at 37 °C in a humid atmosphere of 5% CO_2_. NCM 460 normal colorectal cell was taken as a control.

### 2.9. Cytotoxicity Assay

To assay the cytotoxic effects of these peptides, sacha inchi, curcumin, peptide bioconjugates, and 5-FU, the Alamar Blue HS (high sensitivity) cell viability reagent (St. Louis, MO, USA) was utilized. In all experiments, cells were grown in a 96-well culture-treated plate (BioArrow Techlife, North Point, Hong Kong, China). After being seeded, the cells were exposed to different concentrations (5, 10, 25, 50, 100, 150, and 200 µg/mL) and incubated for 72 h. After the incubation time, 10 µL of the Alamar Blue was added to each well of the plates containing treated cells, untreated cells (negative control), cell culture medium (blank), and incubated for 4 h at 37 °C in a humidified atmosphere of 5% CO_2_. Once incubation was completed, the fluorescence was measured at exc 560 nm and em 590 nm in a Varioskan™ LUX multimode microplate reader (Thermo Fisher Scientific, Waltham, MA, USA). Each experiment was performed in triplicate, and the results were presented as the percentage of metabolic activity using the following equation [8].

% Metabolic Activity = (F treated cells − F blank)/(F untreated cells − F blank) × 100

### 2.10. Selectivity Index (SI)

The selectivity index (SI) was calculated to compare the cytotoxic effect of the compound between non-tumoral and tumoral adherent cell lines. When the SI is higher than 1 indicates desirable selectivity against cancer cells [26].

SI = [(*IC*_50_ no Cancer cells)/(*IC*_50_ Cancer cells)]

### 2.11. Apoptotic Detection via Annexin V-Cy3/6CFDA Assay

To detect apoptosis, Annexin V-Cy3 from Sigma-Aldrich (St. Louis, MO, USA) was used according to the manufacturer’s instructions. An amount of 50 μL of cell line (10,000 cell/well) was seeded in 96-well culture-treated plates (BioArrow Techlife, North Point, Hong Kong, China) for 24 h. After, cells were treated with 50 μL of the *IC*_50_ of each bioactive peptide and bioconjugates. At the end of incubation time conditions (37 in a humidified atmosphere of 5% CO_2_), 25 μL of the Double-Staining Solution (containing Annexin V-Cy3/6-CFDA) was added to each well. Subsequently, readings were taken every 24, 48, and 72 h at Varioskan™ LUX multimode microplate reader (Thermo Fisher Scientific, Waltham, MA, USA). The excitation/emission was 490/525 nm. DMSO 2.5% was used as a control of apoptotic induction [8].

% Normalized Response = [(Sample Fluorescence − Negative control Fluorescence)/(Positive Fluorescence − Negative control Fluorescence)] × 100

A value > 100% indicates a response exceeding that of the positive control.

### 2.12. Caspases 3/7 and 9 Assay

Using the Caspase-Glo 3/7 and Caspase-Glo 9 kits (Promega, Madison, WI, USA), a luminescence assay was conducted. Briefly, 12.5 µL of the cells (20,000 cells/well) were incubated at 37 °C with 5% CO_2_ for 24 h, followed by incubation at 37 °C with 5% CO_2_ for 24 h with 12.5 µL of each bioactive peptide at *IC*_50_ concentration. After the incubation period, 12.5 µg/mL of each reagent was added to the well. Then, 25 µL of Caspase-Glo 3/7 and Caspase-Glo 9 were added to each well, and the luminescence was measured with Varioskan™ LUX multimode microplate reader (Thermo Fisher Scientific, Waltham, MA, USA) every 24 h [8].

% Apoptosis = [(Sample Luminescence − Sample blank)/(Luminescence untreated cells − Sample blank)] × 100

### 2.13. Data Analysis

The analysis of the data generated was performed using GraphPadPrism 10.5 software (GraphPad Software, Inc., La Jolla, CA, USA). For the metabolic activity, we used normalization and non-linear regression versus response; for the test of apoptosis, we used one-way-ANOVA with *t*-test and two-way-ANOVA with the Dunnett test. Data are presented as the mean with a 95% confidence interval. Statistical significance was defined as follows: not significant (ns), *p* > 0.05; * *p* < 0.05; ** *p* < 0.01; *** *p* < 0.001; **** *p* < 0.0001.

## 3. Results

### 3.1. Design and Characterization of Peptide Fragments of PS2Aa1

The Molecular Biology and Biotechnology (BIOMOL) research group at the University of Santander (UDES) has previously investigated the PS2Aa1 (Mpp46Aa1) protein [6,7,8]. In these studies, promising mutants were identified, such as 3–35 (G257V) and N65 (N92D, K175R, S218G). Molecular dynamics simulations further revealed that residues 57, 92, and 101 consistently participate in interactions with the GPI-anchored protein CD59, with ALA105 displaying strong binding to the GPI-CD59 complex. Based on these findings, the protein was fragmented to study the regions involved in interactions with lipid and membrane receptors.

Breaking down proteins by fragmented peptide segmentation and synthesizing these independently is one of the most widely validated methods for studying protein structure, function, and interaction networks. Synthetic peptides allow the isolation of specific regions and the systematic mapping of binding sites, functional domains, and regulatory motifs using overlapping peptide arrays, fragment libraries, or receptor–ligand interaction analyses [27,28,29]. This approach has successfully identified active domains and important regions of complex proteins; for instance, certain peptide fragments can recapitulate the biological activities of their parent proteins, including modulatory effects in vivo [30]. Recent reviews highlight that peptide-based techniques and protein fragment analysis have emerged as essential techniques to understand and manipulate protein–protein interactions, facilitating SAR analyses, high-throughput screening, and rational design of more efficient analogs [31,32]. Furthermore, synthetic peptides are widely regarded as versatile for drug discovery owing to their high purity, reproducibility, and inclusion of targeted mutations or chemical modifications that promote mechanistic investigation and the development of innovative therapeutic candidates [33,34]. All in all, these studies provide compelling evidence of the necessity of protein fragmentation and peptide synthesis as essential strategies for advanced molecular research.

The following peptides were selected for synthesis and analysis: P102-K113, P102-K113-T104L-G108W, F249-Y259, F249-Y259-R252F-P255M, Y95-Y107, Y95-Y107-N97A-P101L, Y95-Y107-N97L-P101L, Y95-N97D, P101L-P102H, and P102H-T104L.

The physicochemical identification of the PS2-derived peptides (Table 1) shows an evident trend in sequence modification and its projected structural behaviour. The majority of the peptides possess a net positive charge (+1 to +2), a characteristic that has been generally associated with enhanced interaction with either negatively charged biological membranes or cancer-cell surfaces. Thus, F249–Y259 and F249–Y259–R252F–P255M with +2 charge can have higher binding affinity, and the binding affinity of anionic lipid environments may affect cytotoxic response. The computed isoelectric points (pI 8.50–9.99) show that all peptides stay positively charged under physiological pH, which lends credence to their potential applications as membrane-active or receptor-interacting ligands.

The largest pI values are associated with short peptides that have fewer acidic residues, which could change the solubility and aggregation properties of the peptides. The range of molecular weight values is approximately from 1214 to 1568 Da, consistent with short-length peptides (11–13 residues). MW varies linearly with residue number and mutation position but remains between the range typically associated with optimum cell penetration and manageable synthesis yields.

GRAVY index shows a wide range in hydrophobicity. Peptides with negative GRAVY values (such as P102–K113 and F249–Y259) show high hydrophilicity, leading to lower membrane insertion and higher solubility. On the other hand, Y95–Y107–N97L–P101L variants that exhibited a positive GRAVY are more hydrophobic in content and may stabilize the α-helical conformations with membrane attachment or provide enhanced attachment between the structures with the structural predictions.

Indeed, higher aliphatic index values (105–127), which are closer to the hydrophobic peptides, indicate higher thermal stability and, perhaps, more structural rigidity, supporting these distinctions. This is consistent with in silico helical predicted values for these variants.

The peptides were synthesized by solid-phase synthesis using an Fmoc/tBu protection strategy. The resulting products were characterized by high-performance liquid chromatography (HPLC) and mass spectrometry (M/S). In all cases, the experimental molecular masses (*m*/*z*) matched the expected theoretical values (Table 1).

**Figure 1:**
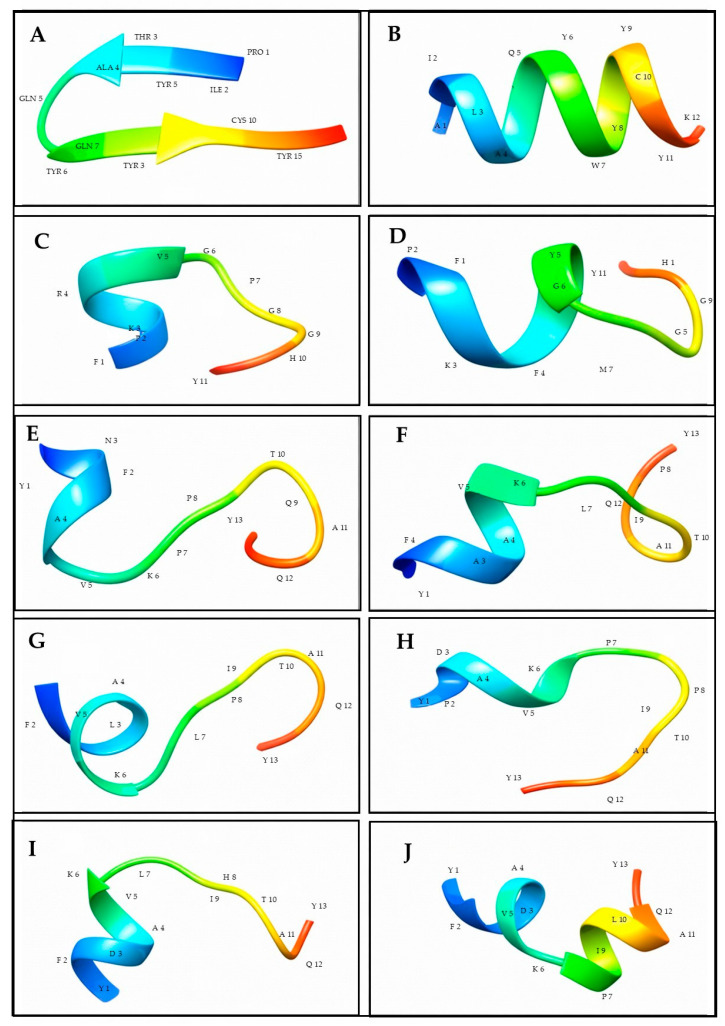
Theoretical in silico secondary structure of peptides. Simulated secondary structure of peptides by PepFOLD 3.5 and visualized by Chimera, where (**A**) P102-K113 (5550), (**B**) T104L-G108W (5551), (**C**) F249-Y259 (5552), (**D**) R252F-P255M (5553), (**E**) Y95-Y107 (5554), (**F**) N97A-P101L (5555), (**G**) N97L-P101L (5556), (**H**) Y95-N97D (5557), (**I**) P101L-P102H (5558), (**J**) P102H-T104L (5559).

The predictions of in silico peptide secondary structure (Figure 1) show that the PS2-produced peptides possess α-helical segments, β-turns, and flexible coil regions, which favor short helices at the central portion, and disordered conformations at the N- and C-terminal. This structural aspect is characteristic of short bioactive molecules, which also need conformational agility for membrane interactions or for binding to a receptor.

**Figure 2:**
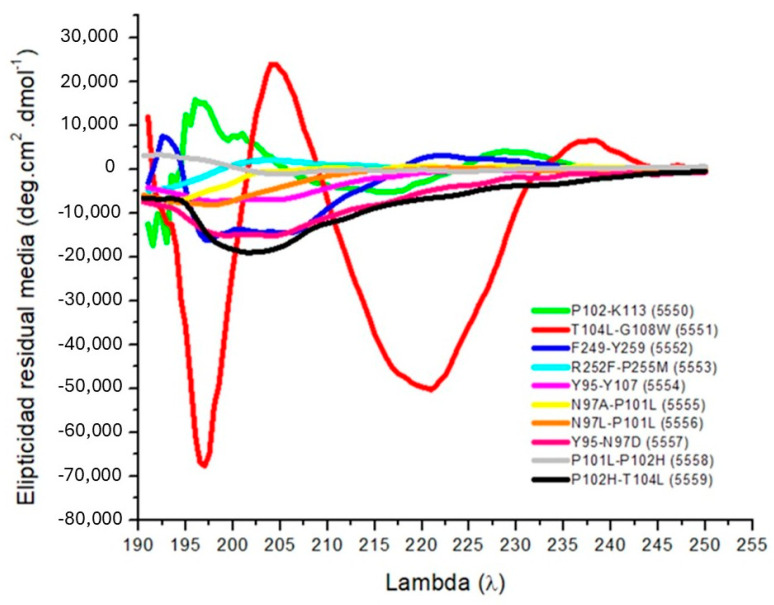
Secondary structure of peptides confirmed by circular dichroism. Circular dichroism spectra of peptides in TFE (30% *w*/*v*). A quartz cell with a path length of 1 mm was used, between 190 and 250 nm at 50 nm/min, with a bandwidth of 0.5 nm. Peptide concentration: 1 mg/mL. CD of P102-K113 up to P102H-T104L.

In comparison with the experimental CD spectra (Figure 2), there is an obvious match between prediction models and solution-phase conformations. Peptide examples include T104L–G108W, where a CD spectrum confirms an α-helical conformation, and shows a high negative ellipticity minimum around 208 nm and another, larger, deeper band at 222 nm. This profile is entirely consistent with its in silico model (Panel B), which models a continuous helix with multiple residues. This stable helical segment, perhaps through insertion in the membrane and certain binding interactions, is thus consistent with the predicted behavior and accounts for the mechanism of biological activity of the peptide.

By contrast, peptides such as P102–K113 or Y95–Y107 display CD spectra characterized primarily by shallow minima and a nearly random-coil signature, with the result that their structure in solution is largely disordered. These experimental findings are consistent with their in silico designs (Figure 1E,G), which predict elongated, flexible structures without a lot of helical configuration. These peptides might potentially depend on induced-fit forces when targeting biological substrates, reflecting their flexible backbone and low intrinsic secondary structure. There are also intermediate behaviors observed. Peptides P101L–P102H (5558) and P102H–T104L (5559) are of modest negative ellipticity at about 208 nm but are deficient in the prominent double minimum observed in stabilized helices, indicating transiently populated, weakly populated helical conformations. This accords with their in silico structures (Figure 1F,J), where partial helices associate with turn and loop regions.

In silico models, on the other hand, often show highly idealized, low-energy configurations that may not account for the full spectrum of conformational diversity in the solution. However, qualitative agreement for most peptides further supports the prediction value of the computational models.

### 3.2. Hemolytic and Cytotoxicity Activity of Peptides

**Figure 3:**
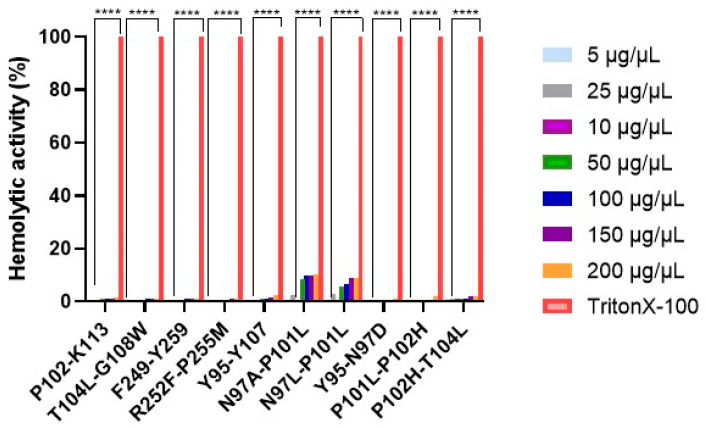
Hemolytic activity of peptides derived from PS2Aa1. Hemolytic activity against sheep red blood cells at different concentrations (5–200 µg/µL). Experiments were performed in three independent replicates. One-way-ANOVA nonparametric tests were performed. The parameters were **** *p* < 0.0001.

The hemolytic assay of the peptides was determined on sheep erythrocytes. The peptides evaluated exhibited a hemolysis percentage less than 11%, which was significantly lower than that of the positive control (TX-100 0.1%), as shown in both Figure 3 and Table 2. The results suggest that all the peptides tested have a promising profile as selective anticancer agents, as they showed no hemotoxicity at the concentrations evaluated.

**Figure 4:**
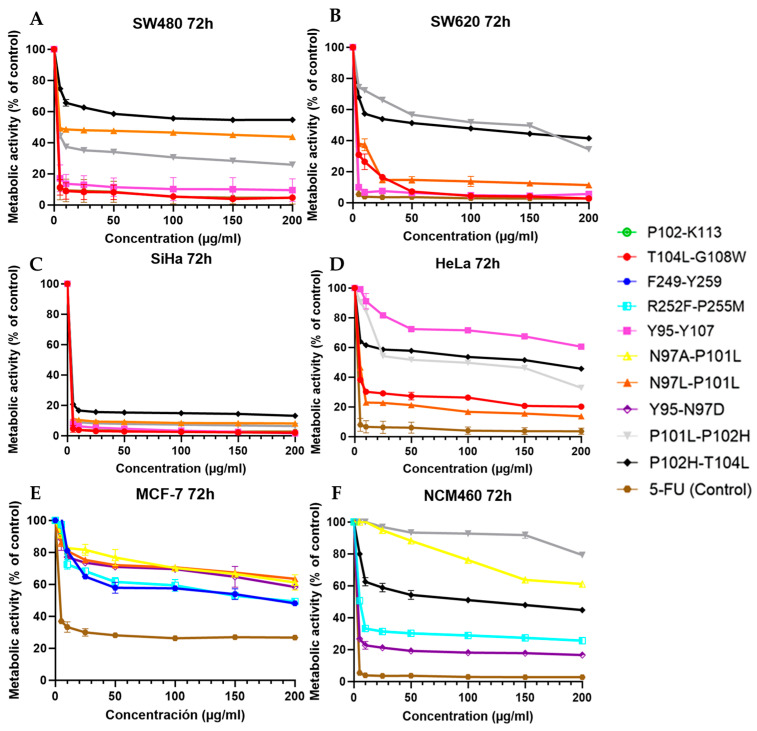
Cytotoxic activity of the most active peptides in each cell line. Normal and cancer cell lines (**A**) SW480, (**B**) SW620, (**C**) SiHa, (**D**) HeLa, (**E**) MCF-7 and, (**F**) NCM460 were treated with peptides and 5-FU at concentrations ranging from 5 to 200 µg/mL. All experiments were performed in three independent replicates. The figures present only the most active peptides for each cell line, displayed using their respective colors and graphical representations. Compounds that are not shown did not exhibit activity in any of the evaluated cell lines.

Moreover, metabolic activity profiles after 72 h show clear differential cytotoxic responses from PS2-derived peptide variants in the colorectal (SW480, SW620), cervical (SiHa, HeLa), and breast (MCF-7) cancer cell lines versus the non-tumoral NCM460. In general, the peptides T104L–G108W, N97A–P101L, and N97L–P101L were consistently superior for dose-dependent reductions in metabolic activity, often reaching values of <20% at concentrations ≥ 50 μg/mL. Hence, these variants are classified as the most potent analogs, with cytotoxic efficiencies comparable to or in some cell lines better than 5-FU, especially in SW620 or SiHa. Specifically, in 2 colorectal cancer models (SW480, Figure 4A, SW620, Figure 4B), representing the primary and metastatic phenotypes, T104L–G108W released its characteristic α-helical conformant profile to maintain an evident and stable decrease in the metabolic activity, even at low concentrations.

Some helical peptides may undergo greater membrane disruption and receptor interaction, maybe accounting for the high level of cytotoxicity that occurred. Likewise, N97A–P101L and N97L–P101L mutants exhibit high activity, in part due to their enhanced hydrophobicity and increased aliphatic index, supporting the notion that hydrophobic enrichment favors adhesion or membrane disruption.

For cervical cancers, SiHa data (Figure 4C) show that virtually all hydrophobic or helical-rich peptides result in a reduction in metabolic activity to below 10%, which suggests that this line is particularly sensitive to a PS2 analog. In HeLa cells (Figure 4D), despite the similar observed cytotoxicity, the relative reduction in the peptides Y95–Y107 and Y95–N97D can be attributed to their excessive disorder and reduced hydrophobicity, allowing them to have weaker interactions with HeLa membrane components or intracellular regulatory targets. When compared with other cancer lines, breast cancer cells MCF-7 present a response to each peptide in a unique manner (Figure 4E) of moderate cytotoxicity, attributed to peptides R252F–P255M and F249–Y259, in contrast to the low potency of peptides in other cancer lines.

This may indicate a receptor or pathway-specific effect in MCF-7 not observed in colorectal or cervical models. The robust activity of T104L–G108W remains, and this also highlights its broad-spectrum activity. Behavioral observation of NCM460 cells (Figure 4F) is important for selectivity estimation. Many of the peptides have cytotoxicity towards non-tumoral cells, and the selective capacity is low at high concentration (among those, T104L–G108W and N97A–P101L). However, Y95–N97D and P101L–P102H variants exhibit significantly reduced toxicity in NCM460 while maintaining activity in cancer lines, indicating a more favorable therapeutic window and pointing to them as promising candidates for further research endeavors.

These patterns demonstrate the significance of structural properties such as helicity, hydrophobic enrichment, and mutation-mediated stabilization in explaining the anticancer activity of PS2-derived peptides. The association between CD-derived helical content and cytotoxic potency, in particular T104L–G108W, suggests that the stability of these conformational components contributes to their function in vivo. In contrast, more commonly, the peptides having the most disorder or low hydrophobic index appear to exhibit more benign or cell-type-restricted activity. This indicates that T104L–G108W, N97A–P101L, and N97L–P101L are the best broad-spectrum candidates, whereas Y95–N97D may be a more selective variant with lower toxicity toward non-tumoral cells. This integrated structural–functional analysis constructs a rational basis to select the PS2-derived peptides for further biological assays or therapeutic optimization.

The additive application of Sacha inchi oil with Parasporin-2-derived peptides had a significant increase in anticancer activity in most cancer cells assayed (SW480, SW620, SiHa, HeLa, and MCF-7). A decrease in metabolic activity was more profound and faster after the addition of the Sacha inchi than peptide-only treatment, demonstrating a synergistic, or at least additive, play between the two bioactive elements.

**Figure 5:**
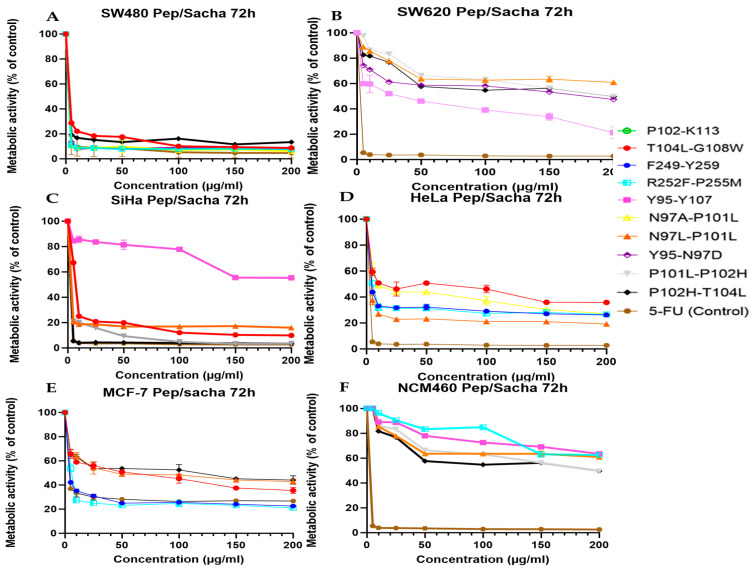
Cytotoxic activity of the most active peptides synergizes with Sacha inchi for each cell line. Normal and cancer cell lines (**A**) SW480, (**B**) SW620, (**C**) SiHa, (**D**) HeLa, (**E**) MCF-7 and, (**F**) NCM460 were treated with peptides and 5-FU at different concentrations, from 5 to 200 µg/µL. Experiments were performed in three independent replicates. The figures present only the most active peptides for each cell line, displayed using their respective colors and graphical representations. Compounds that are not shown did not exhibit activity in any of the evaluated cell lines.

This is in agreement with the evidence of antiproliferative effects of Sacha inchi, due to its high omega-3 fatty acids and antioxidant ingredients that can affect membrane fluidity, induce oxidative stress, or sensitize cancer cells to cytotoxic compounds. The positive synergistic effect was most pronounced in colorectal cancer cells (SW480 and SW620; Figure 5A,B) with peptides T104L–G108W, N97A–P101L, and N97L–P101L, which are already associated with substantial cytotoxic effects alone. In combination with Sacha inchi, these peptides lowered their metabolic activity to almost the basal levels at concentrations of fewer than 50 µg/mL. This points to the fact that polyunsaturated fatty acids induce membrane disruption to facilitate the entry of peptides into the cell or their interactions with membrane proteins, which enhances their cytotoxic actions.

This effect was particularly significant in SW480, where virtually all the peptides showed increased potency in the presence of the oil. For SiHa and HeLa cervical cancer cells (Figure 5C,D), the increase was dependent on the peptide. The peptides that exhibited modest activity, as described above, in isolation (e.g., R252F–P255M, F249–Y259), displayed significant reductions in metabolic activity under shared methods, reaching concentrations comparable to those of their more potent analogs.

This implies Sacha inchi could be used as a sensitizer in cervical cancer models, which could take its toll by causing oxidative imbalance or by disrupting lipid rafts, an important component of a signal to save a cell. Of particular interest was the failure to show strong synergy with Y95–Y107, as this variant, which was only weakly active on its own, implies that this variant has structural or biochemical restrictions and that additional enhancement is not possible. Synergy was also present, although the extent of peptide differed in MCF-7 breast cancer cells (Figure 5E). The hydrophobic and most structurally stable peptides (T104L–G108W, N97A–P101L, and, for instance, N97L–P101L) observed the greatest increase in activity. This combination was shown to have a clear therapeutic superiority over 5-FU.

These findings are consistent with the hypothesis that breast cancer cells may be especially susceptible to treatment approaches that concurrently target membrane integrity and intracellular stress pathways. It is noteworthy that the synergistic effect in the non-tumoral colon cell line NCM460 (Figure 5F) was much decreased. The reduction in metabolic activities of most peptides during co-treatment was quite modest, and it remained above 50% viability at several doses. Such a differential response indicates that normal cells have less sensitivity to the co-action of these PS2 peptides and Sacha inchi, potentially suggesting a therapeutic window and selective cytotoxicity to cancer.

In summary, these findings illustrate the fact that combining the PS2 protein-derived peptides with Sacha inchi potentiates the drugs against various types of cancer, yet does not have a direct influence on healthy patient cells. The observed synergy is probably due to complementary mechanisms: Sacha inchi may enhance membrane permeability, alter redox balance, or perturb survival signaling, to enhance peptide-induced membrane disruptibility or apoptotic pathways. Thus, this two-component approach provides a competitive approach to the discovery and development of natural and peptide-based anticancer therapies.

**Figure 6:**
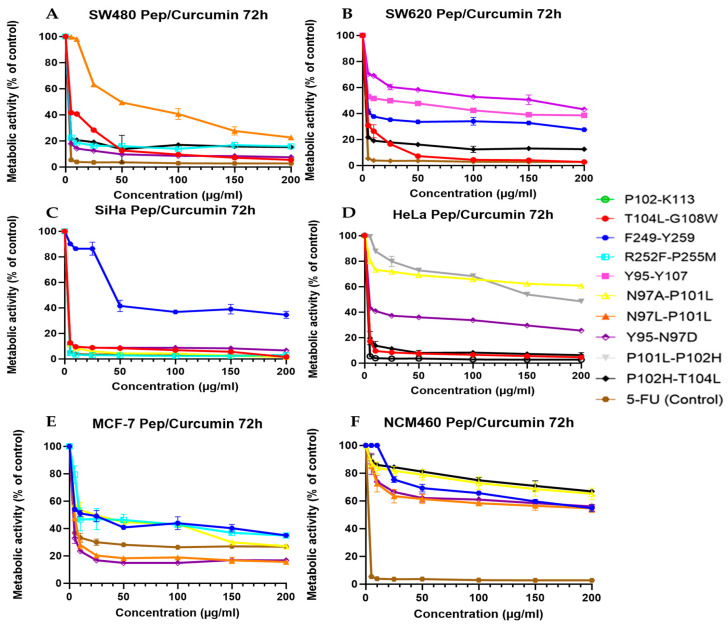
Cytotoxic activity of the most active bioconjugate peptides with curcumin in each cell line. Normal and cancer cell lines (**A**) SW480, (**B**) SW620, (**C**) SiHa, (**D**) HeLa, (**E**) MCF-7 and, (**F**) NCM460 were treated with peptides and 5-FU at different concentrations (5–200 µg/µL). Experiments were performed in three independent replicates. The figures present only the most active peptides for each cell line, displayed using their respective colors and graphical representations. Compounds that are not shown did not exhibit activity in any of the evaluated cell lines.

Conjugation of curcumin with PS2-derived peptides demonstrated significantly higher activity when studied against cancer cell lines. Curcumin by itself is reported to have antioxidant, pro-apoptotic, and anti-inflammatory properties, but has poor solubility and cellular uptake. Its bioactivity seems to be dramatically enhanced when curcumin is covalently or non-covalently linked with small PS2-derived peptides, indicating that PS2-derived peptides are likely to act as delivery enhancers or structural adjunctive promoters, supporting curcumin interaction towards cellular membranes and intracellular targets. Among the colorectal cancer lines (SW480, SW620; Figure 6A,B), the most potent conjugates were T104L–G108W, N97A–P101L, and N97L–P101L, and these reduced metabolic activity to near 0–20%, even at low concentrations.

The peptides showed a strong cytotoxicity by themselves, but when curcumin was linked, the action was strengthened, showing an adjuvant inactivation or supra-additive action. The facilitation may be related to an enhanced intracellular delivery via peptide binding and/or to a synergistic effect of apoptotic and membrane-disruptive mechanisms. In SiHa cervical cancer cells (Figure 6C), the F249–Y259 conjugate inhibited cancer cells to a significant degree that did not occur in the peptide-only or curcumin-only treatment, which could signify a promising functional potential of curcumin conjugation in some of them.

Meanwhile, T104L–G108W, N97A–P101L, and N97L–P101L were also highly inhibitory, indicating their consistent action in cancer models. The results of the HeLa (Figure 6D) cells were heterogeneous for the peptide-induced curcumin conjugation. Moderate hydrophobic peptides (R252F–P255M and F249–Y259) showed modest improvement, while the most structurally stable helical peptides (T104L–G108W) showed marked metabolic inhibition. The more muted response of Y95–Y107 confirms the point that sequence composition and intrinsic structural properties play a significant role in curcumin’s functional potentiation. The MCF-7 breast cancer line (Figure 6E) appeared to benefit most from curcumin-conjugated peptides and also showed significantly greater potency than peptide-only treatment.

Activities attained as high or better than those reported in 5-FU for the most active variants, therefore confirming the potential of peptide conjugation to overcome curcumin’s naturally occurring bioavailability limits. The best performance of the peptides with the higher aliphatic index and favorable folding (e.g., N97L–P101L, N97A–P101L) is consistently exhibited, suggesting that structural compactness and hydrophobic interactions are important for the intracellular retention and activity of curcumin. Also, the NCM460 cells (Figure 6F), which were non-tumoral, had much higher metabolic activity than cancer cells under the same treatment. This could indicate that curcumin–peptide conjugates retain a certain degree of selectivity, so that targeting the malignant cells is more efficient than targeting normal cells.

Y95–N97D, P101L–P102H, and P102H–T104L peptides exhibited selectivity profiles that were particularly well-fitted for cancer cell viability, and the normal cell viability was less affected (Table 3). These results indicate that, while curcumin facilitates apoptosis or oxidative stress pathways in addition to PS2-derived peptides as ligands to cell membrane interaction, cell internalization, and/or cellular structure destabilization at the local level, the effects of peptide–curcumin conjugates may be complementary. The combination results in a more robust and consistent anticancer effect than any function in isolation. This discovery of peptide types with increased potency and maintained selectivity highlights the possibility of these conjugates as anticancer candidates for mechanistic and in vivo research (Table 4 and Table 5).

The bioconjugation of peptides with curcumin significantly improved their cytotoxic activity (Table 5). The T104L-G108W peptide maintained a potent *IC*_50_ of 0.5 μg/mL and showed enhanced cytotoxicity against the MCF-7 cell line. Similarly, the R252F-P255M peptide exhibited strong activity against SiHa and SW480 cells, with an *IC*_50_ of 0.5 μg/mL. The most notable improvement was observed for the Y95-N97D peptide, which demonstrated a substantial increase in potency across all tested cell lines, with *IC*_50_ values ranging from 0.5 to 6.93 μg/mL. In contrast, one peptide showed the highest *IC*_50_ value (92.54 μg/mL), indicating the lowest cytotoxicity (Table 3 and Table 5).

The improvement of cytotoxic activity after bioconjugation of PS2 peptides with curcumin is also mechanistically supported by its combined and complementary biological activities. Curcumin is well established as a pleiotropic anticancer drug that has been found to regulate various cellular pathways, such as induction of apoptosis, mitochondrial dysfunction, and redox imbalance, as well as inhibition of pro-survival signaling cascades, while also having relatively modest bioavailability when treated alone [35,36]. There are several known restrictions regarding curcumin, such as solubilization, cellular uptake, and intracellular delivery, which can be alleviated through peptide bioconjugation, which can achieve higher cytotoxicity [37,38].

In this regard, the low *IC*_50_ value (0.5 μg/mL) was maintained and significant cytotoxicity on the MCF-7 of T104L–G108W–curcumin could indicate that this peptide fragment is an efficient vector for the delivery of curcumin, which might facilitate membrane interaction and intracellular accumulation. Cancer cell membranes, especially in breast cancer, present changes in lipid composition and surface charge, which may favour the interaction of amphipathic peptide–curcumin conjugates, thus enhancing cytotoxic outcomes [39,40]. Likewise, the robust activity of the R252F–P255M conjugate against SiHa and SW480 cells suggests that peptide sequence and local physicochemical properties are critically involved in how cell-type-specific responses are activated after conjugation.

The Y95–N97D–curcumin conjugate showed improved activity with *IC*_50_ values of 0.5–6.93 μg/mL observed in all cancer cell lines. This wide-spectrum improvement suggests curcumin conjugation not only strengthens the peptidical cytotoxicity internally, but it permits multi-target interaction for interleaving mediating membrane destabilization of the peptide backbone with intracellular signaling disruption to its action by being curcumin [36,37]. This dual-action property is an emerging highlight of peptide–small molecule conjugates for anticancer therapy.

Conversely, the highest *IC*_50_ value of 92.54 μg/mL was found in the Y95–N97D peptide, which indicates that the conjugation of curcumin does not itself guarantee better cytotoxicity and also that unfavorable peptide sequences may hinder cell-to-cell interaction, internalization, or curcumin release. These results emphasize the necessity of sequence-dependent design and biophysical compatibility in the peptide–curcumin conjugates and offer confirmation of the need for rational peptide selection to realize the therapeutic capabilities of curcumin-based bioconjugation strategies [38,41]. In general, these findings demonstrate that the curcumin bioconjugation can significantly enhance the anticancer activity of purposively selected PS2-derived peptides, while maintaining or enhancing their potency at low concentrations. This confirms peptide–curcumin conjugates as a platform to create multifunctional anticancer agents that have potential in action, and further mechanistic and in vivo studies are needed.

In contrast, when combined with sacha inchi, a generally antagonistic effect was observed, reducing the cytotoxic activity of most peptides. A notable exception was the P102H-T104L peptide, which showed enhanced cytotoxicity in four out of the five cancer cell lines compared to the peptide alone (Table 3 and Table 4).

Conversely, the mostly antagonistic action found for the addition of PS2-derived peptides with Sacha inchi oil could be biologically justified and could be attributed to various non-mutually exclusive mechanisms related to bioavailability, redox modulation, and membrane dynamics. Sacha inchi oil is rich in polyunsaturated fatty acids (PUFAs), particularly α-linolenic acid, and antioxidant compounds (tocopherols, phenolic compounds), that significantly affect cellular redox homeostasis, membrane properties, and cellular function [42,43,44]. Lipid-rich matrices can also sequester amphipathic molecules by partitioning them into oil droplets or micellar structures to decrease their freely bioavailable fraction of peptides in the aqueous culture medium and to suppress their apparent cytotoxic activity [41,45].

Furthermore, the robust antioxidant activity of Sacha inchi oil might partially counteract oxidative stress-dependent effects on peptide-induced cytotoxicity. Several anticancer peptides and peptide-derived toxins exert part of their activity through redox imbalance, mitochondrial perturbation, or ROS amplification; therefore, potent lipid-soluble antioxidants can dampen these downstream effects, resulting in an antagonistic interaction in combination assays [43,46]. Meanwhile, incorporation of PUFAs into the plasma membrane is known to enhance membrane fluidity and change lipid packing, which can reduce peptide adsorption, pore formation, or internalization for peptides whose mechanism of action relies on stable membrane engagement [40]. These consequences are strongly cell-line dependent and may account for the widely lower cytotoxicity seen in the majority of peptide–Sacha inchi combinations.

The peptide P102H–T104L was an obvious outlier (i.e., the peptide showed a higher cytotoxicity on four out of five cancer lines compared to the peptide alone). This indicates that sequence-specific biophysical properties, including optimized hydrophobic moments, increased propensity for membrane insertion, and better compatibility with PUFA-enriched membranes, may act on the peptide to induce the destabilization of cancer cell membranes, overcoming lipid-mediated sequestration by selectively destabilizing the cancer cell membranes in the presence of Sacha inchi oil [40,45]. Collectively, these observations suggest that the paradoxical antagonism is not an artifact observed in experiments, but rather reflects fundamental matrix- and membrane-mediated modulation of peptide bioactivity, emphasizing the centrality of lipid context and antioxidant load for the study of peptide–oil combination procedures in vitro.

### 3.3. Selectivity Index

Table 6, Table 7 and Table 8 show the selectivity indices (SI) of the crude peptides against tumor and non-tumor cell lines. Notably, for the T104L-G108W peptide, the SI values were comparable to those of the reference anticancer agent used in this study (5-FU). According to criteria established in the literature, a selectivity index ≥ 2.0 is considered pharmacologically relevant [26,47,48], as it indicates at least two-fold greater cytotoxicity toward tumor cells compared to normal cells. These findings demonstrate selective cytotoxicity in vitro and support further investigation of these peptides as potential therapeutic candidates. However, to move beyond this initial proof of concept toward a viable drug candidate, it is essential to complement these results with additional mechanistic and translational studies. First, expanding the panel of non-tumor cell lines to include hepatocytes, cardiomyocytes, and bone marrow cells would allow for a better assessment of the risk of organ-specific toxicity. Second, selectivity must be validated in more physiologically relevant models, such as three-dimensional tumor spheroids, which better represent the tumor microenvironment and intrinsic drug resistance. Finally, in vivo pharmacokinetic and efficacy–toxicity studies in appropriate animal models are required to determine the true therapeutic index and translate the promising in vitro selectivity into a meaningful preclinical profile. Only through an integrated experimental approach can the therapeutic potential of these peptides for the development of targeted antitumor agents be firmly established.

### 3.4. Apoptotic Activity of Peptides

**Figure 7:**
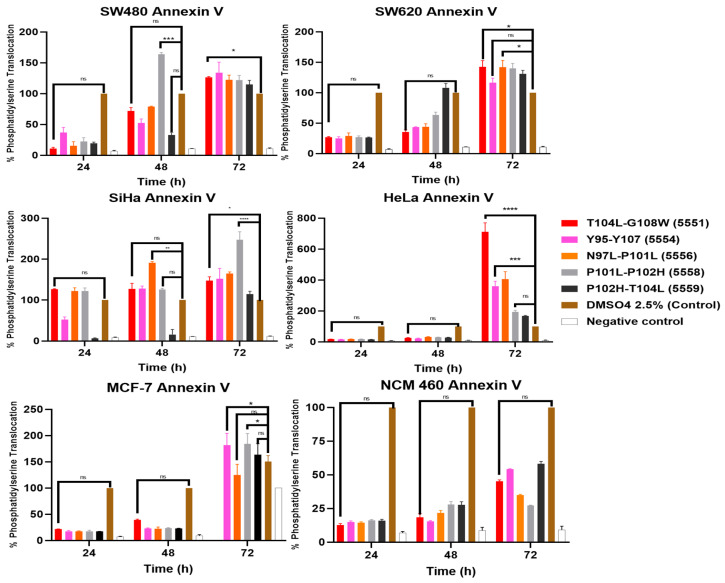
Results of Annexin V/Cy3. Where Annexin V is the graphic corresponding to the translocation of phosphatidylserine after 72 h. Fluorescence data (Annexin V) were normalized as the percentage of apoptotic response relative to the positive control (DMSO 2.5%). Statistical analysis: ns > 0.05, * *p* < 0.05, ** *p* < 0.01, *** *p* < 0.001, **** *p* < 0.0001.

**Figure 8:**
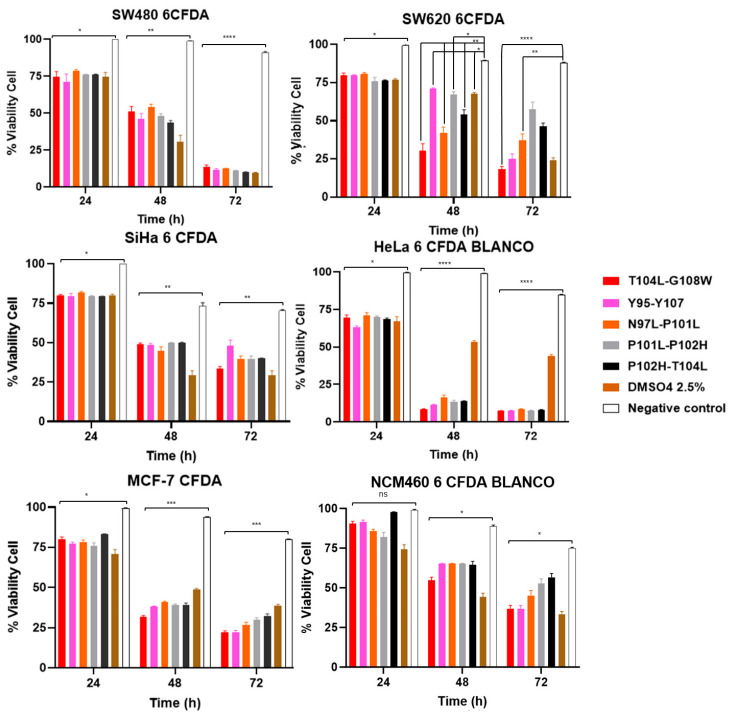
Cell viability by the 6-CFDA assay in SW480, SW620, SiHa, HeLa, MCF-7, and NCM460 cell lines after 24, 48, and 72 h of exposure to Parasporin-2 peptide variants. Bars represent the mean ± SD of three independent experiments. Peptide variants T104L–G108W, Y95–Y107, N97L–P101L, P101L–P102H, and P102H–T104L were evaluated at their corresponding *IC*_50_ concentrations. DMSO (2.5%) was included as an apoptotic induction control, and untreated cells served as the negative control. ns > 0.05, * *p* < 0.05, ** *p* < 0.01, *** *p* < 0.001, **** *p* < 0.0001.

For all cell lines and techniques, the following peptides were used at their corresponding *IC*_50_ concentrations: T104L-G108W, Y95-Y107, N97L-P101L, P101L-P102H, and P102H-T104L. To determine the possible mechanism of cell death caused by the peptides, Annexin V/Cy3 and 6-CFDA were used. This methodology allows for the specific quantification of phosphatidylserine externalization in the plasma membrane, a biochemical event characteristic of the initial stages of apoptosis. The results obtained confirmed the ability of the compounds to induce phosphatidylserine translocation (Figure 7 and Figure 8).

The treatments with crude peptides induced time-dependent increases in the normalized fluorescence signal, expressed as a percentage relative to the positive control. In multiple cases, values exceeding 100% were observed, predominantly in cervical cancer cell lines, indicating that the Annexin V fluorescence recorded for these treatments surpassed that of the positive control (2.5% DMSO). These results are indicative of enhanced phosphatidylserine externalization and, consequently, a more pronounced apoptotic response compared with the control. As the data were obtained by fluorometric quantification using a microplate reader and normalized against internal controls, values above 100% reflect a stronger relative apoptotic response within the dynamic range of the assay rather than an absolute cell count (Table 9).

The fluorescence results obtained from 6-CFDA demonstrated a progressive, time-dependent cytotoxic effect (Figure 8 and Table 10). In the initial stages of treatment, cell viability was approximately 80%, indicating that a substantial proportion of cells retained sufficient metabolic activity to convert 6-CFDA to fluorescein. However, a sustained decrease in fluorescence intensity was observed during the incubation period, reaching values close to 10% at 72 h. This marked reduction reflects a progressive loss of intracellular esterase activity and membrane integrity, consistent with the induction of cell death in later stages. Together with the Annexin V results, these findings support a cumulative cytotoxic effect of the treatment, leading to a significant decrease in cell viability with increasing exposure time.

**Figure 9:**
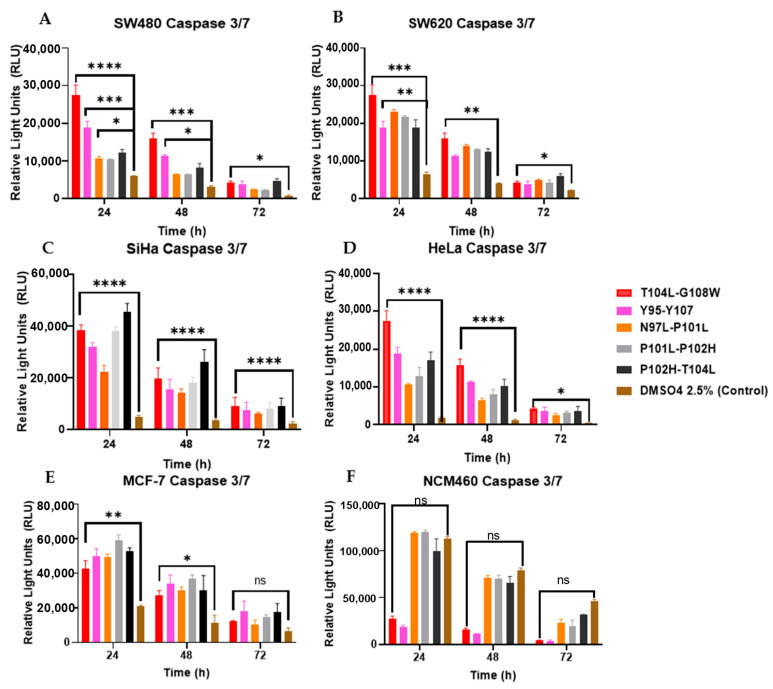
Peptides induce caspase-3/7 activation. Caspase activity was quantified using the Caspase-Glo^®^ 3/7 assay in the following cell lines: (**A**) SW480, (**B**) SW620, (**C**) SiHa, (**D**) HeLa, (**E**) MCF-7, and (**F**) NCM460. No statistically significant differences were observed when compared with the DMSO (2.5%) control. Statistical significance is indicated as follows: ns, *p* > 0.05; * *p* < 0.05; ** *p* < 0.01; *** *p* < 0.001; **** *p* < 0.0001.

**Figure 10:**
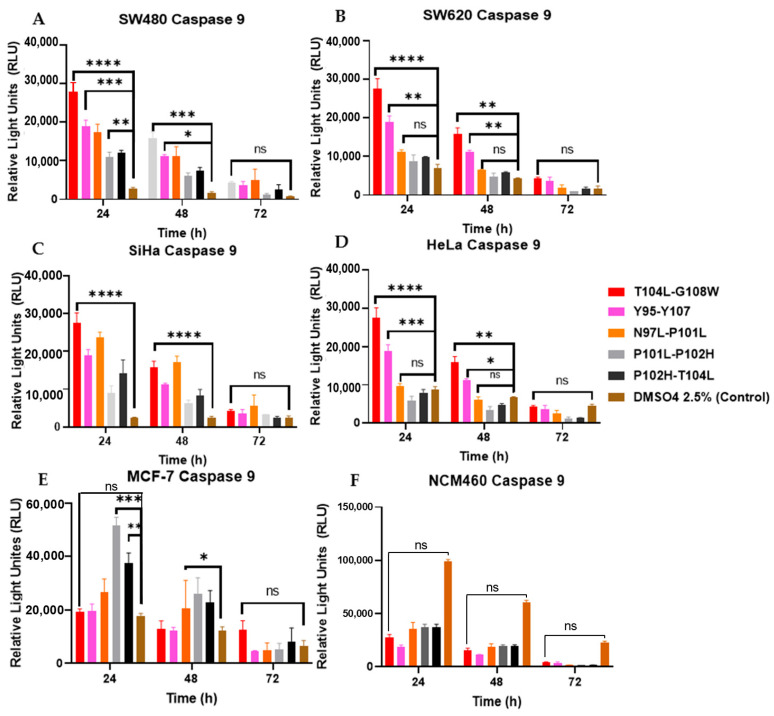
Peptides increase the activation of caspase 9. (**A**) SW480, (**B**) SW620, (**C**) SiHa, (**D**) HeLa, (**E**) MCF-7 and, (**F**) NCM460. Caspase levels were measured using the Caspase-Glo^®^ 9 kits. No significant differences were observed compared to the DMSO control. ns > 0.05, * *p* < 0.05, ** *p* < 0.01, *** *p* < 0.001, **** *p* < 0.0001.

To evaluate the induction of apoptosis by the peptides, caspases 3/7 and 9 were measured every 24 h up to 72 h. It was observed that the peptides caused a greater increase in both caspases after 24 h. T104L-G108W was the best peptide for most of the cell lines used (Figure 9 and Figure 10).

## 4. Discussion

Parasporins have emerged as a potential therapeutic agent due to their specific cytotoxic activity [48,49]. The peptides derived from N65 and their synergistic effect with curcumin and Sacha inchi exhibit a growth inhibition of HeLa, SiHa, SW480, SW620, and MCF-7, respectively. These results are similar to those found in studies conducted by our research group, where N65 obtained an *IC*_50_ of 1.2 µg/mL in colorectal cancer cells [19,20]. For breast and cervix cancer cell lines, Brasseur et al. [3]. Reported that the native protein has good activity and decreases metabolic activity at 20 μg/mL with a decrease in 5% and 72%, respectively. The modified peptides showed greater activity compared to the native peptide P102-K113. Among them, T104L-G108W was the most active in all assays (Table 3, Table 4 and Table 5), which means that the structure of the peptide plays a fundamental role in its activity. It has been documented that ACPs defined as α-helix are more effective in disrupting cancerous membranes by forming pores in the cell membrane through the interaction of ligands expressed in cancerous lines. Added to this is the positive charge of the peptides (T104L-G108W/+1) facilitate interaction with the negative membrane of cancer cell lines, promoting electrostatic interactions that lead to cell death [50,51].

The substitution of aromatic or polar residues in anticancer peptides, coupled with the assessment of their [48,49] secondary structure, provides a robust and evidence-based framework for interpreting these outcomes. As reported for amphipathic α-helical peptides, the synergy between net positive charge and amphipathicity enhances their affinity toward negatively charged tumor membranes, promoting insertion, permeabilization, and selectivity over non-malignant cells [52]. This trend was particularly evident in T104L-G108W, which exhibited a well-defined α-helical conformation. Such structural characteristics are consistent with mechanistic models in which amphipathic helices optimize interactions with tumor lipid bilayers, drive mitochondrial depolarization, and, subsequently, activate intrinsic apoptotic pathways [53,54].

Other noteworthy analogs were Y95-Y107, N97L-P101L, P101L-P102H, and P102H-T104L. Furthermore, evaluation of the synergistic effect with curcumin and Sacha inchi revealed increased cytotoxic activity, even in peptides that were initially inactive. A particularly notable case is F249-Y259, which showed marked activity against the MCF-7 cell line when combined with both compounds (Figure 6, Table 5). The differential resistance of antimicrobial or anticancer peptides among different cell lines may be due to variations in the amount of cholesterol found in their membranes. While it is true that cholesterol content is a key modulator of the activity of these peptides, it is crucial to establish the comparison correctly. Contrary to what has been suggested, it has been documented that the SiHa cell line has a higher cholesterol content in its membrane compared to the MCF-7 line. This lipid profile, with a more rigid and orderly membrane in SiHa, creates a more effective permeability barrier. Consequently, this difference allows the activity of the peptides to be contrasted: peptides targeting the plasma membrane would be expected to show reduced activity in the SiHa line (rich in cholesterol) compared to MCF-7, where the membrane is more fluid and susceptible to disruption by peptides [55].

Modified parasporin fragments, particularly variants T104L-G108W, N97L-P101L, and P101L-P102H, demonstrated significant pro-apoptotic activity. Treatment with these peptides induced marked activation of the initiator caspase-9 and the effector caspases-3/7 within 24 h, indicating early triggering of the intrinsic apoptotic pathway [56]. This was followed by the externalization of phosphatidylserine, detectable via Annexin V staining after 72 h (Figure 7). The delay observed between caspase activation and phosphatidylserine exposure is a recognized characteristic of the apoptotic process (Figure 9 and Figure 10). This occurs when activated caspases inactivate flippases and activate scramblases, ultimately leading to the surface exposure of phosphatidylserine as a ‘find-me’ signal for phagocytic cells [57].

In future studies, we plan to incorporate additional pharmacological endpoints to further strengthen the mechanistic understanding of the peptide T104L-G108W and bioactive combinations. In particular, migration assays and clonogenic survival assays will be performed to evaluate long-term proliferative capacity, metastatic potential, and the durability of the observed cytotoxic responses. These complementary approaches will allow a more comprehensive assessment of anticancer activity and will help clarify whether the functional effects observed in the present study translate into sustained inhibition of cancer cell propagation.

## 5. Conclusions

This investigation demonstrates that Parasporin-2-derived peptide–curcumin conjugates exhibit pronounced and reproducible anticancer activity across diverse tumor models while maintaining comparatively low toxicity toward non-malignant cells. Among the evaluated variants, T104L–G108W emerged as the most potent broad-spectrum peptide, consistently achieving significant reductions in metabolic activity and displaying structural features, such as a stable helical core, that likely facilitate efficient membrane interaction and intracellular engagement. In parallel, the selective peptides Y95–N97D, P101L–P102H, and P102H–T104L significantly reduced cancer cell viability with minimal impact on normal epithelial cells, underscoring their therapeutic promise. Together, these findings reveal a complementary mechanistic framework in which curcumin enhances apoptotic and oxidative stress pathways, while PS2-derived peptides promote membrane association, cell internalization, and localized destabilization of cellular structures. The synergistic integration of these mechanisms results in superior anticancer effects compared with either component alone. In summary, this study provides compelling evidence supporting peptide–curcumin conjugates, and particularly T104L–G108W, as strong candidates for continued mechanistic exploration and in vivo validation, thereby advancing the development of next-generation peptide-based anticancer therapeutics.

## Figures and Tables

**Table 1:** Physicochemical properties of PS2Aa1-derived peptides.

Name	Sequence	Residues	Charge	^1^ Pl	MW (Da)	Gravy	Aliphatic Index
P102-K113	PITAXXXXXCYK-NH_2_	12	+1	8.52	1469.67	−0.542	40.83
T104L-G108W	PILXXXWYYCYK-NH_2_	12	+1	8.52	1610.8	−0.208	73.33
F249-Y259	FPKXXXXGGHY-NH_2_	11	+2	9.99	1214.39	−0.936	26.36
R252F-P255M	FPKXXXXXGHY-NH_2_	11	+2	8.60	1239.46	0.045	26.36
Y95-Y107	YFNAVKXXXXXQY-NH_2_	13	+2	8.50	1511.74	−0.177	67.69
N97A-P101L	YFAAVKXXXXXQY-NH_2_	13	+1	8.50	1484.76	0.646	105.38
N97L-P101L	YFLAVXXXXXAQY-NH_2_	13	+1	8.50	1526.84	0.800	127.69
Y95-N97D	YFDAVKXXXXXQY-NH_2_	13	+1	5.83	1512.73	−0.177	67.69
P101L-P102H	YFDAVKXXXXXQY-NH_2_	13	+1	6.74	1568.79	0.115	97.69
P102H-T104L	YFDAVKXXXXXQY-NH_2_	13	+1	6.74	1564.80	0.046	97.69

^1^ Pl (isoelectric point), MW (molecular weight). Where X can be the amino acid sequence of = QYWY, QYGY, VGMG, VGPG, VKLP, VKPP, VKPP, VKLP, VKPH, and VKLH.

**Table 2:** Percentage of HC_50_ of the peptides.

Synthesis Number	Peptide	% HC_50_ at 200 µM
5550	P102-K113	1.2%
5551	T104L-G108W	1.3%
5552	F249-Y259	2.4%
5553	R252F-P255M	10%
5554	Y95-Y107	9.1%
5555	N97A-P101L	1.3%
5556	N97L-P101L	2.1%
5557	Y95-N97D	1.2%
5558	P101L-P102H	1.7%
5559	P102H-T104L	1.9%

**Table 3:** Half-maximal inhibitory concentration (*IC*_50_) of the peptides against all treated cell lines.

Name	Sequence	*IC*_50_ (µg/mL)					
		Cell Line					
		SiHa	SW480	SW620	HeLa	MCF-7	NCM460
P102-K113 (5550)	NH_3_-PITAXXXXXCYK-CONH_2_	112.36 ± 1.2	149.28 ± 0.84	120.53 ± 1.97	80.9 ± 1.38	>200 ± 1.7	>200 ± 4.13
T104L-G108W (5551)	PILXXXWYYCYK-CONH_2_	<5.0 ± 0.76	<5.0 ± 1.2	<5.0 ± 0.89	<5.0 ± 1.72	>200 ± 1.4	>200 ± 7.45
F249-Y259 (5552)	FPKXXXXGGHY-CONH_2_	38.26 ± 1.35	>200 ± 3.5	58.67 ± 1.47	>200 ± 1.41	4.93 ± 1.22	169.2 ± 2.79
R252F-P255M (5553)	FPKXXXXXGHY-CONH_2_	39.42 ± 2.01	33.53 ± 2.3	34.01 ± 1.76	35.5 ± 1.59	>200 ± 5.56	173.5 ± 3.15
Y95-Y107 (5554)	YFNAVKXXXXXQY-CONH_2_	<5.0 ± 0.91	<5.0 ± 0.49	<5.0 ± 0.56	<5.0 ± 0.78	158.9 ± 1.14	>200 ± 1.22
N97A-P101L (5555)	YFAAVKXXXXXQY-CONH_2_	>200 ± 1.56	>200 ± 1.78	>200 ± 1.35	>200 ± 1.28	198.2 ± 1.52	>200 ± 1.57
N97L-P101L (5556)	YFLAVXXXXXAQY-CONH_2_	<5.0 ± 1.11	27.96 ± 2.28	6.24 ± 2.01	11.01 ± 186	187.5 ± 0.94	198.2 ± 3.43
Y95-N97D (5557)	YFDAVKXXXXXQY-CONH_2_	158.5 ± 0.89	138.4 ± 2.36	188.7 ± 1.24	>200 ± 2.93	177.2 ± 1.85	195.1 ± 4.41
P101L-P102H (5558)	YFDAVKXXXXXQY-CONH_2_	<5.0 ± 1.67	24.72 ± 0.78	55.25 ± 0.96	172.2 ± 2.51	>200 ± 3.17	190.2 ± 1.45
P102H-T104L (5559)	YFDAVKXXXXXQY-CONH_2_	<5.0 ± 0.65	166 ± 3.12	64.83 ± 0.63	185.3 ± 1.81	>200 ± 2.77	199.4 ± 1.91
5-FU	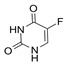	<5.0 ± 0.61	<5.0 ± 1.92	<5.0 ± 1.85	<5.0 ± 1.28	<5.0 ± 1.78	<5.0 ± 1.08

Where X can be the amino acid sequence of = QYWY, QYGY, VGMG, VGPG, VKLP, VKPP, VKPP, VKLP, VKPH, and VKLH.

**Table 4:** Half-maximal inhibitory concentration (*IC*_50_) values of the Sacha inchi with peptides across all evaluated cell lines.

Name	Sequence	*IC*_50_ (µg/mL)					
		Cell Line					
		SiHa	SW480	SW620	HeLa	MCF-7	NCM460
P102-K113 (5550)	NH_3_-PITAXXXXXCYK-CONH_2_	>200 ± 4.16	>200 ± 1.91	>200 ± 1.20	>200 ± 3.16	>200 ± 1.74	>200 ± 2.28
T104L-G108W (5551)	NH_3_-PILXXXWYYCYK-CONH_2_	111.1 ± 1.21	<5.0 ± 1.44	>200 ± 1.15	153.1 ± 4.06	111.1 ± 1.78	>200 ± 2.26
F249-Y259 (5552)	NH_3_-FPKXXXXGGHY-CONH_2_	>200 ± 1.23	187.4 ± 1.64	>200 ± 1.04	162.4 ± 7.14	6.87 ± 0.84	>200 7± 2.31
R252F-P255M (5553)	NH_3_-FPKXXXXXGHY-CONH_2_	<5.0 ± 2.75	172.5 ± 1.73	>200 ± 1.10	175.8 ± 1.84	<5.0 ± 0.75	188.9 ± 2.32
Y95-Y107 (5554)	NH_3_-YFNAVKXXXXXQY-CONH_2_	<5.0 ± 1.32	<5.0 ± 2.21	12.45 ± 1.01	>200 ± 2.18	184.2 ± 1.41	184.6 ± 1.72
N97A-P101L (5555)	NH_3_-YFAAVKXXXXXQY-CONH_2_	184.9 ± 0.96	8.8 ± 1.62	181.5 ± 1.22	46.29 ± 1.41	156.3 ± 3.48	>200 ± 1.37
N97L-P101L (5556)	NH_3_-YFLAVXXXXXAQY-CONH_2_	42.58 ± 1.80	7.6 ± 2.05	53.29 ± 1.13	>200 ± 1.27	188.4 ± 2.01	>200 ± 1.45
Y95-N97D (5557)	NH_3_-YFDAVKXXXXXQY-CONH_2_	55.92 ± 1.16	16.7 ± 2.57	42.21 ± 1.11	>200 ± 2.25	133.2 ± 4.76	>200 ± 1.93
P101L-P102H (5558)	NH_3_-YFDAVKXXXXXQY-CONH_2_	21.42 ± 1.78	43.43 ± 1.48	31.09 ± 1.03	93.21 ± 4.25	195.3 ± 2.77	>200 ± 1.02
P102H-T104L (5559)	NH_3_-YFDAVKXXXXXQY-CONH_2_	<5.0 ± 2.68	<5.0 ± 1.80	6.87 ± 1.24	<5.0 ± 1.54	82.9 ± 3.11	>200 ± 1.95
5-FU	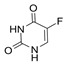	<5.0 ± 1.25	<5.0 ± 1.21	<5.0 ± 1.62	<5.0 ± 3.43	<5.0 ± 1.79	<5.0 ± 1.64

Where X can be the amino acid sequence of = QYWY, QYGY, VGMG, VGPG, VKLP, VKPP, VKPP, VKLP, VKPH, and VKLH.

**Table 5:** Half-maximal inhibitory concentration (*IC*_50_) values of the bioconjugated peptides with curcumin across all evaluated cell lines.

Name	Sequence	*IC*_50_ (µg/mL)					
		Cell Line					
		SiHa	SW480	SW620	HeLa	MCF-7	NCM460
P102-K113 (5550)	NH_3_-PITAXXXXXCYK-CONH_2_	175.6 ± 1.47	>200 ± 1.28	>200 ± 0.55	152.33 ± 1.79	199.35 ± 1.56	>200 ± 1.85
T104L-G108W (5551)	NH_3_-PILXXXWYYCYK-CONH_2_	<5.0 ± 2.12	<5.0 ± 1.58	<5.0 ± 2.97	<5.0 ± 2.44	150.43 ± 4.44	>200 ± 1.43
F249-Y259 (5552)	NH_3_-FPKXXXXGGHY-CONH_2_	48.74 ± 1.77	199.52 ± 0.81	6.40 ± 1.15	195.0 ± 1.92	20.03 ± 1.67	>200 ± 1.77
R252F-P255M (5553)	NH_3_-FPKXXXXXGHY-CONH_2_	<5.0 ± 2.05	<5.0 ± 1.52	199.05 ± 2.44	134.7 ± 2.02	7.73 ± 0.77	>200 ± 1.22
Y95-Y107 (5554)	NH_3_-YFNAVKXXXXXQY-CONH_2_	158.48 ± 1.21	82.87 ± 1.16	19.27 ± 2.76	158.64 ± 1.61	>200 ± 1.85	>200 ± 2.88
N97A-P101L (5555)	NH_3_-YFAAVKXXXXXQY-CONH_2_	12.18 ± 1.62	62.11 ± 1.73	131.09 ± 0.88	46.26 ± 1.24	46.73 ± 2.67	>200 ± 1.43
N97L-P101L (5556)	NH_3_-YFLAVXXXXXAQY-CONH_2_	100 ± 1.52	50.47 ± 1.52	160.28 ± 1.5	>200 ± 2.99	6.77 ± 1.56	>200 ± 1.24
Y95-N97D (5557)	NH_3_-YFDAVKXXXXXQY-CONH_2_	6.93 ± 3.37	<5.0 ± 1.02	92.54 ± 1.34	<5.0 ± 1.57	<5.0 ± 1.05	>200 ± 1.27
P101L-P102H (5558)	NH_3_-YFDAVKXXXXXQY-CONH_2_	162.84 ± 4.74	160.25 ± 1.94	177.82 ± 2.44	125.62 ± 2.52	>200 ± 1.67	>200 ± 2.28
P102H-T104L (5559)	NH_3_-YFDAVKXXXXXQY-CONH_2_	158.91 ± 1.01	<5.0 ± 2.41	<5.0 ± 1.99	<5.0 ± 1.42	>200 ± 1.24	>200 ± 1.55
5-FU	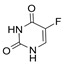	<5.0 ± 1.36	<5.0 ± 0.82	<5.0 ± 1.39	<5.0 ± 1.66	<5.0 ± 1.90	<5.0 ± 1.72

Where X can be the amino acid sequence of = QYWY, QYGY, VGMG, VGPG, VKLP, VKPP, VKPP, VKLP, VKPH, and VKLH.

**Table 6:** Selectivity index of crude peptide against all the treated cells.

Name	Sequence	Selectivity Index (SI)					
		Cell Line					
		SiHa	SW480	SW620	HeLa	MCF-7	NCM460
P102-K113 (5550)	NH_3_-PITAXXXXXCYK-CONH_2_	1.7	1.0	1.0	1.3	1.0	1.0
T104L-G108W (5551)	NH_3_-PILXXXWYYCYK-CONH_2_	40.0	40.0	40.0	40.0	1.3	1.0
F249-Y259 (5552)	NH_3_-FPKXXXXGGHY-CONH_2_	4.10	1.0	31.25	1.0	10	1.0
R252F-P255M (5553)	NH_3_-FPKXXXXXGHY-CONH_2_	40.0	40.0	1.0	1.5	25.6	1.0
Y95-Y107 (5554)	NH_3_-YFNAVKXXXXXQY-CONH_2_	1.26	2.4	10.4	1.7	1.0	1.0
N97A-P101L (5555)	NH_3_-YFAAVKXXXXXQY-CONH_2_	16.42	3.2	1.5	4.3	4.3	1.0
N97L-P101L (5556)	NH_3_-YFLAVXXXXXAQY-CONH_2_	20.0	4.0	1.2	1.0	29.4	1.0
Y95-N97D (5557)	NH_3_-YFDAVKXXXXXQY-CONH_2_	29.0	40.0	2.2	40.0	40.0	1.0
P101L-P102H (5558)	NH_3_-YFDAVKXXXXXQY-CONH_2_	1.2	1.2	1.1	1.2	1.0	1.0
P102H-T104L (5559)	NH_3_-YFDAVKXXXXXQY-CONH_2_	1.3	40.0	40.0	40.0	1.0	1.0
5-FU	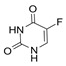	40.0	40.0	40.0	40.0	40.0	40.0

Where X can be the amino acid sequence of = QYWY, QYGY, VGMG, VGPG, VKLP, VKPP, VKPP, VKLP, VKPH, and VKLH.

**Table 7:** Selectivity index of Sacha inchi peptide against all the treated cells.

Name	Sequence	Selectivity Index (SI)					
		Cell Line					
		SiHa	SW480	SW620	HeLa	MCF-7	NCM460
P102-K113 (5550)	NH_3_-PITAXXXXXCYK-CONH_2_	1.0	1.0	1.0	1.0	1.0	1.0
T104L-G108W (5551)	NH_3_-PILXXXWYYCYK-CONH_2_	1.8	40.0	1.0	1.4	1.8	1.0
F249-Y259 (5552)	NH_3_-FPKXXXXGGHY-CONH_2_	1.0	1.1	1.0	1.2	29.1	1.0
R252F-P255M (5553)	NH_3_-FPKXXXXXGHY-CONH_2_	40.0	1.5	1.0	1.5	40.0	1.0
Y95-Y107 (5554)	NH_3_-YFNAVKXXXXXQY-CONH_2_	40.0	40.0	16.4	1.0	1.0	1.0
N97A-P101L (5555)	NH_3_-YFAAVKXXXXXQY-CONH_2_	1.1	22.7	1.1	4.3	1.3	1.0
N97L-P101L (5556)	NH_3_-YFLAVXXXXXAQY-CONH_2_	4.6	4.0	1.2	1.0	29.4	1.0
Y95-N97D (5557)	NH_3_-YFDAVKXXXXXQY-CONH_2_	3.57	40.0	2.2	40.0	40.0	1.0
P101L-P102H (5558)	NH_3_-YFDAVKXXXXXQY-CONH_2_	9.33	11.3	4.73	1.0	1.0	1.0
P102H-T104L (5559)	NH_3_-YFDAVKXXXXXQY-CONH_2_	40.0	40.0	40.0	3.5	1.0	1.0
5-FU	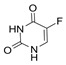	40.0	40.0	40.0	40.0	40.0	40.0

Where X can be the amino acid sequence of = QYWY, QYGY, VGMG, VGPG, VKLP, VKPP, VKPP, VKLP, VKPH, and VKLH.

**Table 8:** Selectivity index of bioconjugated peptide with curcumin against all the treated cells.

Name	Sequence	Selectivity Index (SI)					
		Cell Line					
		SiHa	SW480	SW620	HeLa	MCF-7	NCM460
P102-K113 (5550)	NH_3_-PITAXXXXXCYK-CONH_2_	1.14	1.0	1.0	1.3	1.0	1.0
T104L-G108W (5551)	NH_3_-PILXXXWYYCYK-CONH_2_	40.0	40.0	40.0	40.0	1.4	1.0
F249-Y259 (5552)	NH_3_-FPKXXXXGGHY-CONH_2_	4.1	1.0	31.25	1,1	10	1.0
R252F-P255M (5553)	NH_3_-FPKXXXXXGHY-CONH_2_	40.0	40.0	1.0	1.5	26	1.0
Y95-Y107 (5554)	NH_3_-YFNAVKXXXXXQY-CONH_2_	1.3	2.4	10.4	1.3	1.0	1.0
N97A-P101L (5555)	NH_3_-YFAAVKXXXXXQY-CONH_2_	16.4	3.2	1.5	4.3	4.2	1.0
N97L-P101L (5556)	NH_3_-YFLAVXXXXXAQY-CONH_2_	2	3.92	1.3	1.0	30.0	1.0
Y95-N97D (5557)	NH_3_-YFDAVKXXXXXQY-CONH_2_	29.0	40.0	2,16	40.0	40.0	1.0
P101L-P102H (5558)	NH_3_-YFDAVKXXXXXQY-CONH_2_	1.22	1.2	1.1	1.6	1.0	1.0
P102H-T104L (5559)	NH_3_-YFDAVKXXXXXQY-CONH_2_	1.3	40.0	40.0	40.0	1.0	1.0
5-FU	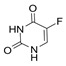	40.0	40.0	40.0	40.0	40.0	40.0

Where X can be the amino acid sequence of = QYWY, QYGY, VGMG, VGPG, VKLP, VKPP, VKPP, VKLP, VKPH, and VKLH.

**Table 9:** Percentage of Annexin V-positive cells determined by fluorescence.

Name	Sequence	Time (h)	Percentages					
			Cell Line					
			SiHa	SW480	SW620	HeLa	MCF-7	NCM460
T104L-G108W (5551)	PILXXXWYYCYK-CONH_2_	24	128.4%	13%	28%	18%	17%	11%
		48	129%	69%	35%	20%	29%	18%
		72	140%	122%	144%	700%	61%	46%
Y95-Y107 (5554)	YFNAVKXXXXXQY-CONH_2_	24	45.8%	30%	28%	18%	15%	12%
		48	129%	52%	38%	20%	17%	12%
		72	142%	131%	125%	343%	162%	52%
N97L-P101L (5556)	YFLAVXXXXXAQY-CONH_2_	24	125.4%	15%	30%	18%	15%	12%
		48	190%	77%	38%	20%	17%	19%
		72	122%	120%	144%	375%	140%	34%
P101L-P102H (5558)	YFDAVKXXXXXQY-CONH_2_	24	125.4%	20%	28%	18%	15%	13%
		48	129%	166%	60%	20%	17%	27%
		72	240%	120%	142%	184%	162%	27%
P102H-T104L (5559)	YFDAVKXXXXXQY-CONH_2_	24	8.9%	19%	28%	18%	15%	13%
		48	11%	25%	110%	20%	17%	27%
		72	110%	117%	132%	162%	154%	55%
DMSO	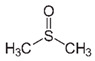	24	100%	100%	100%	100%	100%	100%
		48	100%	100%	100%	100%	100%	100%
		72	100%	100%	100%	100%	100%	100%
Negative control (Medium)	N/A	24	9%	5%	5%	6%	8%	6%
		48	9%	8%	9%	7%	9%	7%
		72	9%	10%	11%	8%	100%	7%

Where X can be the amino acid sequence of = QYWY, QYGY, VGMG, VGPG, VKLP, VKPP, VKPP, VKLP, VKPH, and VKLH.

**Table 10:** Percentage of viable cells quantified by 6-CFDA fluorescence.

Name	Sequence	Time (h)	Percentages					
			Cell Line					
			SiHa	SW480	SW620	HeLa	MCF-7	NCM460
T104L-G108W (5551)	PILXXXWYYCYK-CONH_2_	24	128.4%	13%	28%	18%	17%	11%
		48	129%	69%	35%	20%	29%	18%
		72	140%	122%	144%	700%	61%	46%
Y95-Y107 (5554)	YFNAVKXXXXXQY-CONH_2_	24	45.8%	30%	28%	18%	15%	12%
		48	129%	52%	38%	20%	17%	12%
		72	142%	131%	125%	343%	162%	52%
N97L-P101L (5556)	YFLAVXXXXXAQY-CONH_2_	24	125.4%	15%	30%	18%	15%	12%
		48	190%	77%	38%	20%	17%	19%
		72	122%	120%	144%	375%	140%	34%
P101L-P102H (5558)	YFDAVKXXXXXQY-CONH_2_	24	125.4%	20%	28%	18%	15%	13%
		48	129%	166%	60%	20%	17%	27%
		72	240%	120%	142%	184%	162%	27%
P102H-T104L (5559)	YFDAVKXXXXXQY-CONH_2_	24	8.9%	19%	28%	18%	15%	13%
		48	11%	25%	110%	20%	17%	27%
		72	110%	117%	132%	162%	154%	55%
DMSO	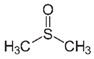	24	100%	100%	100%	100%	100%	100%
		48	100%	100%	100%	100%	100%	100%
		72	100%	100%	100%	100%	100%	100%
Negative control (Medium)	N/A	24	9%	5%	5%	6%	8%	6%
		48	9%	8%	9%	7%	9%	7%
		72	9%	10%	11%	8%	100%	7%

Where X can be the amino acid sequence of = QYWY, QYGY, VGMG, VGPG, VKLP, VKPP, VKPP, VKLP, VKPH, and VKLH.

## Data Availability

The original contributions presented in this study are included in the article/Supplementary Material. Further inquiries can be directed to the corresponding author.

## Supplementary Materials

The following supporting information can be downloaded at: https://www.mdpi.com/article/10.3390/cancers18030451/s1, Table S1: Quality parameters of the P102-K113 (5550) peptide models; Table S2: Quality parameters of the T104L-G108W(5551) peptide models; Table S3: Quality parameters of the F249-Y259(5552) peptide models; Table S4: Quality parameters of the R252F-P255M (5553) peptide models; Table S5: Quality parameters of the Y95-Y107 (5554) peptide models; Table S6: Quality parameters of the N97A-P101L (5555) peptide models; Table S7: Quality parameters of the N97L-P101L (5556) peptide models; Table S8: Quality parameters of the Y95-N97D (5557) peptide models; Table S9: Quality parameters of the P101L-P102H (5558) peptide models; Table S10: Quality parameters of the P102L-T104L (5559) peptide models; Figure S1: Characterization of P102-K113 (5550) peptide. (A) Chromatogram spectra. (B) Mass spectra; Figure S2: Characterization of T104L-G108W (5551) peptide. (A) Chromatogram spectra. (B) Mass spectra; Figure S3: Characterization of F249-Y259 (5552) peptide. (A) Chromatogram spectra. (B) Mass spectra; Figure S4: Characterization of R252F-P255M (5553) peptide. (A) Chromatogram spectra. (B) Mass spectra; Figure S5: Characterization of Y95-Y107 (5554) peptide. (A) Chromatogram spectra. (B) Mass spectra; Figure S6: Characterization of N97A-P101L (5555) peptide. (A) Chromatogram spectra. (B) Mass spectra; Figure S7: Characterization of N97L-P101L (5556) peptide. (A) Chromatogram spectra. (B) Mass spectra; Figure S8: Characterization of Y95-N97D (5557) peptide. (A) Chromatogram spectra. (B) Mass spectra; Figure S9: Characterization of P101L-P102H (5558) peptide. (A) Chromatogram spectra. (B) Mass spectra and, Figure S10: Characterization of P102H-T104L (5559) peptide. (A) Chromatogram spectra. (B) Mass spectra.

## Abbreviations

The following abbreviations are used in this manuscript:

λ	Wavelength
*Bt*	*Bacillus thuringiensis*
PS	Parasporins
TFP	Pore-forming toxins
GPI	Glycosylphosphatidylinositol
APN	Aminopeptidase N
HBTU	2-(1H-benzotriazol-1-yl)-1,1,3,3-tetramethyluronium hexafluorophosphate
HCTU	2-(6-Chloro-1-H-benzotriazol-1-yl)-1,1,3,3-tetramethylammonium hexafluorophosphate
TBTU	2-(1H-Benzotriazole-1-yl)-1,1,3,3-tetramethylammonium tetrafluoroborate
DMF	N,N-dimethylformamide
DIPEA	N,N-diisopropylethylamine
TFA	Trifluoroacetic acid
TIS	Triisopropylsilane
RP-HPLC	Reverse-phase high-performance liquid chromatography
AI	Artificial intelligence
CD	Circular dichroism
DMEM	Dulbecco’s modified eagle medium
FBS	Fetal bovine serum
PS-2	Parasporin-2
HC_50_	Hemolytic concentration
*IC*_50_	Half-maximal inhibitory concentration

## References

[1] Bukowski K., Kciuk M., Kontek R. (2020). Mechanisms of multidrug resistance in cancer chemotherapy. Int. J. Mol. Sci..

[2] Bray F., Laversanne M., Sung H., Ferlay J., Siegel R.L., Soerjomataram I., Jemal A. (2024). Global cancer statistics 2022: GLOBOCAN estimates of incidence and mortality worldwide for 36 cancers in 185 countries. CA Cancer J. Clin..

[3] Brasseur K., Auger P., Asselin E., Parent S., Côté J.C., Sirois M. (2015). Parasporin-2 from a new *Bacillus thuringiensis* 4R2 strain induces caspases activation and apoptosis in human cancer cells. PLoS ONE.

[4] Dong Z., Zhang X., Zhang Q., Tangthianchaichana J., Guo M., Du S., Lu Y. (2024). Anticancer mechanisms and potential anti-cancer applications of antimicrobial peptides and their nano agents. Int. J. Nanomed..

[5] Amano H., Yamagiwa M., Akao T., Mizuki E., Ohba M., Sakai H. (2005). A Novel 29-kDa crystal protein from *Bacillus thuringiensis* induces caspase activation and cell death of Jurkat T cells. Biosci. Biotechnol. Biochem..

[6] Cruz J., Suárez-Barrera M.O., Rondón-Villarreal P., Olarte-Diaz A., Guzmán F., Visser L., Rueda-Forero N.J. (2021). Computational study, synthesis and evaluation of active peptides derived from Parasporin-2 and spike protein from Alphacoronavirus against colorectal cancer cells. Biosci. Rep..

[7] Suárez-Barrera M.O., Visser L., Pinzón-Reyes E.H., Rondón Villarreal P., Alarcón-Aldana J.S., Rueda-Forero N.J. (2022). Site-Directed Mutants of Parasporin PS2Aa1 with enhanced cytotoxic activity in colorectal cancer cell lines. Molecules.

[8] Alarcón-Aldana J.S., Visser L., Rueda-Forero N.J., Pinzón-Reyes E.H., Rondón-Villarreal P., Suárez-Barrera M.O. (2024). Enhancing the cytotoxicity and apoptotic efficacy of Parasporin-2-derived variants (Mpp46Aa1) on cancer cell lines. Toxins.

[9] Unlu A., Nayir E., Kalenderoglu M.D., Kirca O., Ozdogan M. (2016). Curcumin (Turmeric) and cancer. JBUON.

[10] Wang W., Li M., Wang L., Chen L., Goh B.C. (2023). Curcumin in cancer therapy: Exploring molecular mechanisms and overcoming clinical challenges. Cancer Lett..

[11] Vargas J., Arbelaez N., Cardenas D., Murillo J., Ospina V., Robledo S., Soto J. (2025). In vitro antitumor capacity of extracts obtained from the plants *Plukenetia volubilis* (*Sacha inchi*) and *Moringa oleifera* in gastric cancer. F1000Research.

[12] Lelle M., Kaloyanova S., Freidel C., Theodoropoulou M., Musheev M., Niehrs C., Stalla G., Peneva K. (2015). Octreotide-mediated tumor-targeted drug delivery via a cleavable doxorubicin-peptide conjugate. Mol. Pharm..

[13] Currie J.C., Demeule M., Charfi C., Zgheib A., Larocque A., Danalache B.A., Ouanouki A., Béliveau R., Marsolais C., Annabi B. (2022). The peptide-drug conjugate TH1902: A new sortilin receptor-mediated cancer therapeutic against ovarian and endometrial cancers. Cancers.

[14] Zhu Y.S., Tang K., Lv J. (2021). Peptide-drug conjugate-based novel molecular drug delivery system in cancer. Trends Pharmacol. Sci..

[15] Saghaeidehkordi A., Chen S., Yang S., Kaur K. (2021). Evaluation of a keratin 1 targeting peptide-doxorubicin conjugate in a mouse model of triple-negative breast cancer. Pharmaceutics.

[16] Heh E., Allen J., Ramirez F., Lovasz D., Fernandez L., Hogg T., Riva H., Holland N., Chacon J. (2023). Peptide drug conjugates and their role in cancer therapy. Int. J. Mol. Sci..

[17] Yang S.B., Banik N., Han B., Lee D.N., Park J. (2022). Peptide-based bioconjugates and therapeutics for targeted anticancer therapy. Pharmaceutics.

[18] Agrawal P., Bhagat D., Mahalwal M., Sharma N., Raghava G.P.S. (2021). AntiCP 2.0: An updated model for predicting anticancer peptides. Brief. Bioinform..

[19] Rathore A.S., Choudhury S., Arora A., Tijare P., Raghava G.P.S. (2024). ToxinPred 3.0: An improved method for predicting the toxicity of peptides. Comput. Biol. Med..

[20] Kaur A., Pati P.K., Pati A.M., Nagpal A.K. (2020). Physico-chemical characterization and topological analysis of pathogenesis-related proteins from *Arabidopsis thaliana* and *Oryza sativa* using in-silico approaches. PLoS ONE.

[21] Virtanen P., Gommers R., Oliphant T.E., Haberland M., Reddy T., Cournapeau D., Burovski E., Peterson P., Weckesser W., Bright J. (2020). SciPy 1.0: Fundamental algorithms for scientific computing in Python. Nat. Methods.

[22] Lamiable A., Thévenet P., Rey J., Vavrusa M., Derreumaux P., Tufféry P. (2016). PEP-FOLD3: Faster de novo structure prediction for linear peptides in solution and in complex. Nucleic Acids Res..

[23] Waterhouse A., Bertoni M., Bienert S., Studer G., Tauriello G., Gumienny R., Heer R., Beer T.A., Rempfer C., Bordoli L. (2018). SWISS-MODEL: Homology modelling of protein structures and complexes. Nucleic Acids Res..

[24] Pettersen E.F., Goddard T.D., Huang C.C., Couch G.S., Greenblatt D.M., Meng E.C., Ferrin T.E. (2004). UCSF Chimera—A visualization system for exploratory research and analysis. J. Comput. Chem..

[25] Guzmán F., Gauna A., Roman T., Luna O., Álvarez C., Pareja-Barrueto C., Albericio F., Cárdenas C. (2021). Tea bags for Fmoc solid-phase peptide synthesis: An example of circular economy. Molecules.

[26] Peña-Morán O.A., Villarreal M.L., Álvarez-Berber L., Meneses-Acosta A., Rodríguez-López V. (2016). Cytotoxicity, Post-treatment recovery, and selectivity analysis of naturally occurring podophyllotoxins from *Bursera fagaroides* var. fagaroides on breast cancer cell lines. Molecules.

[27] Santa-Coloma T.A. (2022). Overlapping synthetic peptides as a tool to map protein-protein interactions FSH as a model system of nonadditive interactions. Biochim. Biophys. Acta Gen. Subj..

[28] Volkmer R., Tapia V., Landgraf C. (2012). Synthetic peptide arrays for investigating protein interaction domains. FEBS Lett..

[29] Savinov A., Fernandez A., Fields S. (2022). Mapping functional regions of essential bacterial proteins with dominant-negative protein fragments. Proc. Natl. Acad. Sci. USA.

[30] Meyer K., Selbach M. (2020). Peptide-based interaction proteomics—PubMed. Moll. Cell. Proteom..

[31] Beza J., Bedoya M., Cruz P., Ojeda P., Adasme-Carreño F., Cerda O., González W. (2025). Main methods and tools for peptide development based on protein-protein interactions (PPIs). Biochem. Biophys. Res. Commun..

[32] Gupta S., Azadvari N., Hosseinzadeh P. (2022). Design of protein segments and peptides for binding to protein targets. BioDesign Res..

[33] Cárdenas C., Santana P., Álvarez C., Mercado L., Marshall S., Albericio F., Guzmán F. (2024). Synthetic peptides as valuable and versatile tools for research: Our 20 year journey in Chile. Explor. Drug Sci..

[34] Zhong X., Xie Y., Chen Y., Lu Y., Hou M. (2025). Recent progress in peptide-based fluorescent probes biomedical applications: A review. Int. J. Nanomed..

[35] Aggarwal B.B., Harikumar K.B. (2009). Potential therapeutic effects of curcumin, the anti-inflammatory agent, against neurodegenerative, cardiovascular, pulmonary, metabolic, autoimmune and neoplastic diseases. Int. J. Biochem. Cell Biol..

[36] Kunnumakkara A.B., Bordoloi D., Padmavathi G., Monisha J., Roy N.K., Prasad S., Aggarwal B.B. (2017). Curcumin, the golden nutraceutical: Multitargeting for multiple chronic diseases. Br. J. Pharmacol..

[37] Crintea A., Motofelea A.C., Șovrea A.S., Constantin A.M., Crivii C.B., Carpa R., Duțu A.G. (2023). Dendrimers: Advancements and potential applications in cancer diagnosis and treatment—An overview. Pharmaceutics.

[38] Gupta S., Kapoor P., Chaudhary K., Gautam A., Kumar R., Raghava G.P.S. (2013). In silico approach for predicting toxicity of peptides and proteins. PLoS ONE.

[39] Hoskin D.W., Ramamoorthy A. (2008). Studies on anticancer activities of antimicrobial peptides. Biochim. Biophys. Acta.

[40] Habault J., Poyet J.L. (2019). Recent Advances in Cell Penetrating Peptide-Based Anticancer Therapies. Molecules.

[41] Roell K.R., Reif D.M., Motsinger-Reif A.A. (2017). An introduction to terminology and methodology of chemical synergy—Perspectives from across disciplines. Front. Pharmacol..

[42] Chirinos R., Zuloeta G., Pedreschi R., Mignolet E., Larondelle Y., Campos D. (2013). Sacha inchi (*Plukenetia volubilis*) seed oil: Fatty acid composition, tocopherols, phenolic compounds, and antioxidant capacity. Food Chem..

[43] Ruiz A., Ortega-Jácome J.F., Mora J.R., Landázuri A.C., Vásconez Duchicela P., Vásconez-Espinoza J., Beltrán-Ayala P., Andrade-Cuvi M.J., Alvarez-Suarez J.M. (2025). Comprehensive characterization and valorization potential of Amazonian *Sacha inchi* (*Plukenetia volubilis* L.) seeds, oil, and oilcake by-products for sustainable food applications. Front. Nutr..

[44] Redjeki S.G., Hulwana A.F., Aulia R.N., Maya I., Chaerunisaa A.Y., Sriwidodo S. (2025). Sacha Inchi (*Plukenetia volubilis*): Potential Bioactivity, Extraction Methods, and Microencapsulation Techniques. Molecules.

[45] Caesar L.K., Cech N.B. (2019). Synergy and antagonism in natural product extracts: When 1 + 1 does not equal 2. Nat. Prod. Rep..

[46] Uti D.E., Atangwho I.J., Alum E.U., Ntaobeten E., Obeten U.N., Bawa I., Agada S.A., Ukam C.I., Egbung G.E. (2025). Antioxidants in cancer therapy mitigating lipid peroxidation without compromising treatment through nanotechnology. Discov. Nano.

[47] Oliveira P.F., Alves J.M., Damasceno J.L., Oliveira R.A.M., Dias H.J., Crotti A.E.M., Crispim D. (2015). Cytotoxicity screening of essential oils in cancer cell lines. Rev. Bras. Farmacogn..

[48] Lica J.J., Wieczór M., Grabe G.J., Heldt M., Jancz M., Misiak M., Gucwa K., Brankiewicz W., Maciejewska N., Stupak A. (2021). Effective Drug Concentration and Selectivity Depends on Fraction of Primitive Cells. Int. J. Mol. Sci..

[49] Kitada S., Abe Y., Maeda T., Shimada H. (2009). Parasporin-2 requires GPI-anchored proteins for the efficient cytocidal action to human hepatoma cells. Toxicology.

[50] Fahs S., Patil-Sen Y., Snape T.J. (2015). Foldamers as anticancer therapeutics: Targeting protein-protein interactions and the cell membrane. Chembiochem. Eur. J. Chem. Biol..

[51] López-Vallejo F., Caulfield T., Martínez-Mayorga K., Giulianotti M.A., Nefzi A., Houghten R.A., Medina-Franco J.L. (2011). Integrating virtual screening and combinatorial chemistry for accelerated drug discovery. Comb. Chem. High Throughput Screen..

[52] Chiangjong W., Chutipongtanate S., Hongeng S. (2020). Anticancer peptide: Physicochemical property, functional aspect and trend in clinical application (Review). Int. J. Oncol..

[53] Campanile M., Oliva R., D’Errico G., Vecchio P.D., Petraccone L. (2023). The anticancer peptide LL-III alters the physico-chemical properties of a model tumor membrane promoting lipid bilayer permeabilization. Phys. Chem. Chem. Phys..

[54] Barragán-Cárdenas A.C., Insuasty-Cepeda D.S., Cárdenas-Martínez K.J., López-Meza J., Ochoa-Zarzosa A., Umaña-Pérez A., Rivera-Monroy Z., García-Castañeda J. (2022). LfcinB-derived peptides: Specific and punctual change of an-amino acid in monomeric and dimeric sequences increase selective cytotoxicity in colon cancer cell lines. Arab. J. Chem..

[55] Todor I.N., Lukyanova N.Y., Chekhun V.F. (2012). The lipid content of cisplatin- and doxorubicin-resistant MCF-7 human breast cancer cells. Exp. Oncol..

[56] Rezaeidian J., Naseh V., Entezari M., Ziyadi H., Hashemi M. (2024). Curcumin induces MCF-7 breast cancer cell apoptosis through miR-15a alteration. Int. J. Cancer Manag..

[57] Elmore S. (2007). Apoptosis: A review of programmed cell death. Toxicol. Pathol..

