# Supplementary material for: Parasporin-2-Derived Peptide Fragments: Characterization and Synergistic Anticancer Activity with Sacha Inchi and Curcumin
Source: Cancers (Basel). 2026 Jan 30;18(3):451. doi: 10.3390/cancers18030451 (PMC12897068; doi:10.3390/cancers18030451)

cancers

MDPI

Supplementary Materials

Scoring of peptides generated by Swiss model evaluation. The results are shown below.

P102-K113 (5550)

PITAXXXXXCYK-NH_2

Table S1. Quality parameters of the P102-K113 (5550) peptide models.

Parameters	Model 1	Model 2	Model 3	Model 4	Model 5
Molprobity Score	0.50	1.88	1.40	0.50	1.40
Clash Score	0	5.10	0	0	0
Favored Ramachandran	100%	87.50%	62.50%	100%	62.50%
Ramanchandra outliers	0	0	25%	0	0
Rotamer outliers	0	0	0	0	0
C-Beta deviations	0	0	0	0	0
Incorrect bonds	0/104	0/104	0/104	0/104	0/104
Incorrect angles	0/137	0/137	0/137	0/137	0/137

T104L-G108W(5551)

PILXXXWYYCYK-NH_2

Table S2. Quality parameters of the T104L-G108W(5551) peptide models.

Parameters	Model 1	Model 2	Model 3	Model 4	Model 5
Molprobity Score	0.50	0.50	0.50	0.50	0.50
Clash Score	0	0	0	0	0
Favored Ramachandran	100%	100%	100%	100%	100%
Ramanchandra outliers	0	0	0	0	0
Rotamer outliers	0	0	0	0	0
C-Beta deviations	0	0	1	0	0
Incorrect bonds	0/117	0/117	0/117	0/117	0/117
Incorrect angles	0/156	0/156	0/156	0/156	0/156

F249-Y259(5552)

FPKXXXXGGHY-NH_2

Table S3. Quality parameters of the F249-Y259(5552) peptide models.

Parameters	Model 1	Model 2	Model 3	Model 4	Model 5
Molprobity Score	1.29	1.40	1.40	1.53	1.29
Clash Score	0	0	0	0	0
Favored Ramachandran	75%	62.50%	62.50%	37.50%	75.0%
Ramanchandra outliers	12.50%	12.50%	12.50%	12.50%	12.50%
Rotamer outliers	0	0	0	0	0
C-Beta deviations	0	0	0	0	0
Incorrect bonds	2/88	1/88	1/88	1/88	1/88
Incorrect angles	1/116	1/116	2/116	2/116	2/116

R252F-P255M (5553)

FPKXXXXXGHY-NH_2

Table S4. Quality parameters of the R252F-P255M (5553) peptide models.

Parameters	Model 1	Model 2	Model 3	Model 4	Model 5
Molprobity Score	1.93	2.23	2.11	1.47	1.40
Clash Score	5.85	0	5.85	0	0
Favored Ramachandran	87.50%	62.50%	75%	50%	62.50%
Ramanchandra outliers	0	0	12.50%	50%	0
Rotamer outliers	0	12.50%	0	0	0
C-Beta deviations	1	1	0	1	0
Incorrect bonds	1/89	1/89	1/89	1/89	1/89
Incorrect angles	2/116	2/116	0/116	0/116	4/116

Y95-Y107 (5554)

YFNAVKXXXXXQY-NH_2

Table S5. Quality parameters of the Y95-Y107 (5554) peptide models.

Parameters	Model 1	Model 2	Model 3	Model 4	Model 5
Molprobity Score	2.16	2.15	3.08	1.40	1.40
Clash Score	0	4.76	4.76	0	0
Favored Ramachandran	87.50%	62.50%	25%	62.50%	62.50%
Ramanchandra outliers	12.50%	37.50%	25.0%	12.50%	12.50%

Rotamer outliers	10%	0	10%	0	0
C-Beta deviations	1	1	0	1mm	0
Incorrect bonds	0/108	0/108	0/108	0/108	0/108
Incorrect angles	0/145	0/145	0/145	0/145	0/145

N97A-P101L (5555)

YFAAVKXXXXXQY-NH_2

Table S6. Quality parameters of the N97A-P101L (5555) peptide models.

Parameters	Model 1	Model 2	Model 3	Model 4	Model 5
Molprobity Score	2.04	1.29	1.11	1.40	1.11
Clash Score	4.74	0	0	0	0
Favored Ramachandran	75.0%	100%	25%	62.50%	62.50%
Ramanchandra outliers	12.50%	0	25.0%	12.50%	12.50%
Rotamer outliers	0	11.11%	0	0	0
C-Beta deviations	0	0	0	0	0
Incorrect bonds	0/105	0/105	0/105	0/105	0/105
Incorrect angles	0/140	1/140	1/140	1/140	1/140

N97L-P101L (5556)

YFLAVXXXXXAQY-NH_2

Table S7. Quality parameters of the N97L-P101L (5556) peptide models.

Parameters	Model 1	Model 2	Model 3	Model 4	Model 5
Molprobity Score	1.11	1.11	1.11	1.29	0.50
Clash Score	0	0	0	0	0
Favored Ramachandran	87.50%	87.50%	87.50%	75.0%	100%
Ramanchandra outliers	12.50%	12.50%	12.50%	12.50%	0
Rotamer outliers	0	0	0	0	0
C-Beta deviations	0	0	0	0	0
Incorrect bonds	0/108	0/108	0/108	0/108	0/108
Incorrect angles	0/144	1/144	1/144	1/144	1/144

Y95-N97D (5557)

YFDAVKXXXXXQY-NH_2

Table S8. Quality parameters of the Y95-N97D (5557) peptide models.

Parameters	Model 1	Model 2	Model 3	Model 4	Model 5
Molprobity Score	2.05	2.23	3.03	1.29	2.23
Clash Score	0	0	4.81	0	0
Favored Ramachandran	75.0%	50%	75.0%	75.0%	50%
Ramanchandra outliers	25%	37.50%	12.50%	0	37.50%
Rotamer outliers	10%	10%	20%	0	10%
C-Beta deviations	0	0	0	1	1
Incorrect bonds	0/108	0/108	0/108	0/108	0/108
Incorrect angles	0/145	1/145	1/145	1/145	1/145

P101L-P102H (5558)

YFDAVKXXXXXQY-NH_2

Table S9. Quality parameters of the P101L-P102H (5558) peptide models.

Parameters	Model 1	Model 2	Model 3	Model 4	Model 5
Molprobity Score	2.00	1.11	1.87	2.61	0.50
Clash Score	4.63	0	0	4.63	0
Favored Ramachandran	100%	87.50%	87.50%	75.50%	100%
Ramanchandra outliers	25%	37.50%	12.50%	0	37.50%
Rotamer outliers	10%	10%	20%	0	10%
C-Beta deviations	0	0	0	1	1
Incorrect bonds	0/108	0/108	0/108	0/108	0/108
Incorrect angles	0/145	1/145	1/145	1/145	1/145

P102L-T104L (5559)

YFDAVKXXXXXQY-NH_2

Table S10. Quality parameters of the P102L-T104L (5559) peptide models.

Parameters	Model 1	Model 2	Model 3	Model 4	Model 5
Molprobity Score	2.16	1.11	1.87	2.61	0.50
Clash Score	0	0	0	4.63	0
Favored Ramachandran	62.50%	87.50%	87.50%	75.50%	100%
Ramanchandra outliers	0	37.50%	12.50%	0	37.50%

Rotamer outliers	10%	10%	20%	0	10%
C-Beta deviations	0	0	0	1	1
Incorrect bonds	1/112	1/112	1/112	2/112	2/112
Incorrect angles	2/149	1/149	1/149	1/149	1/149

Figure S1. Characterization of P102-K113 (5550) peptide. A) Chromatogram spectra. B) Mass spectra.

A

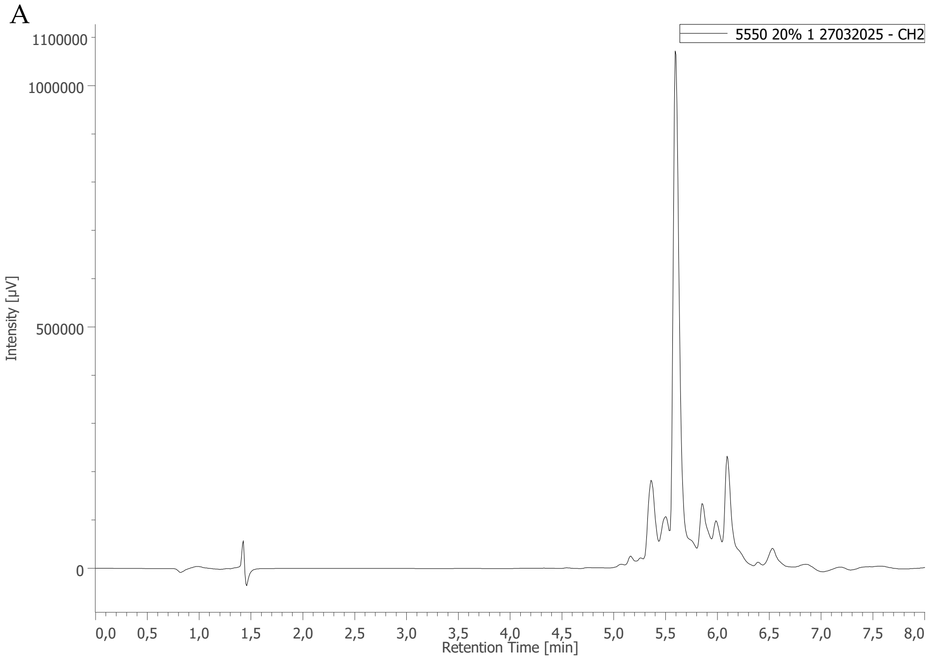

B

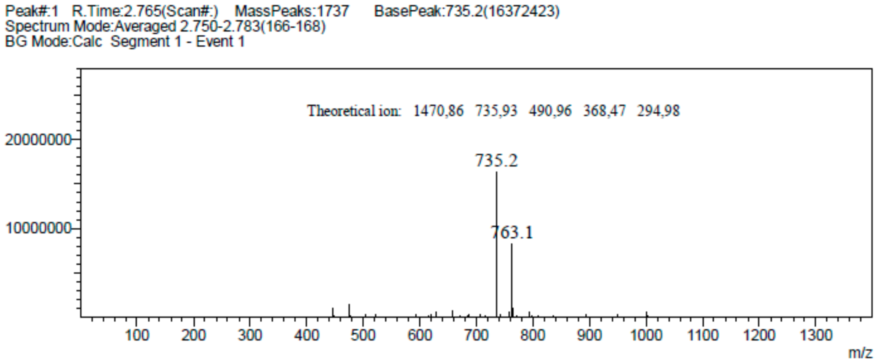

Figure S2. Characterization of T104L-G108W (5551) peptide. A) Chromatogram spectra. B) Mass spectra.

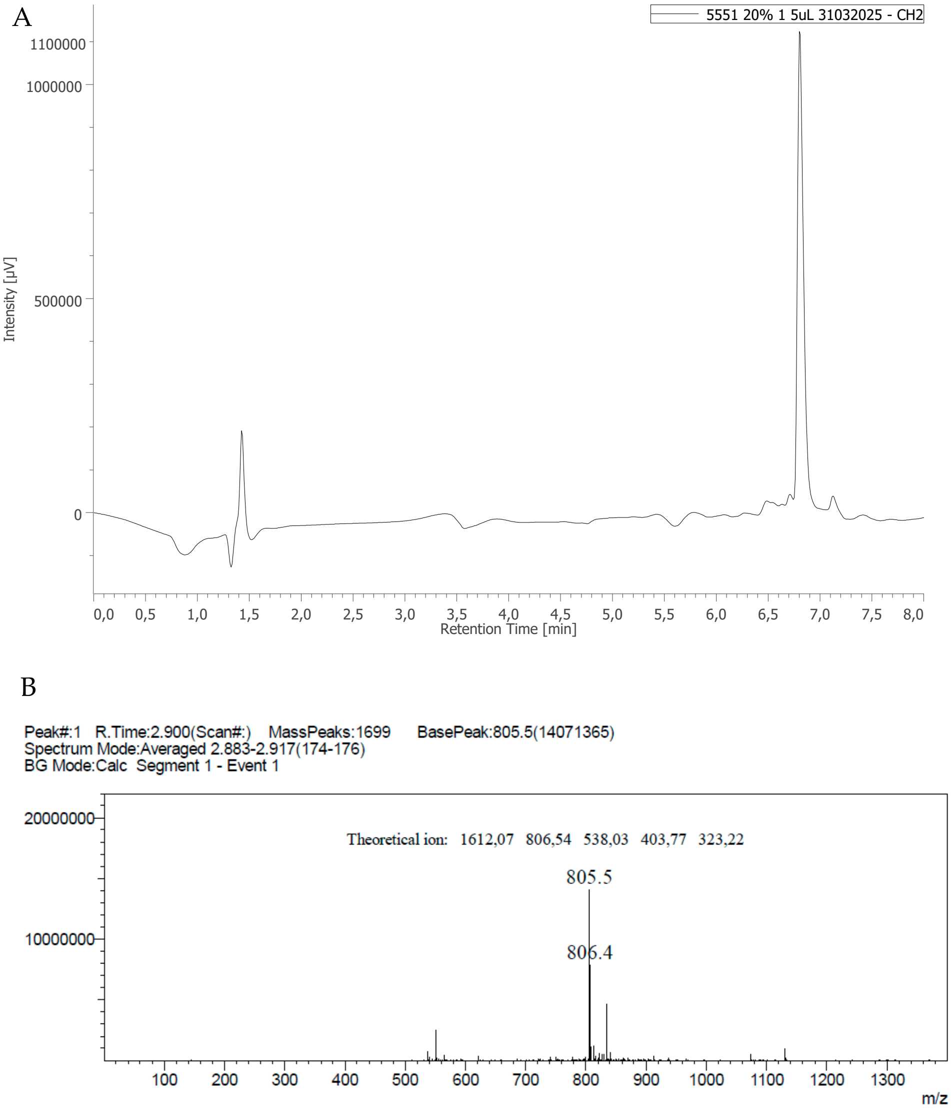

Figure S3. Characterization of F249-Y259 (5552) peptide. A) Chromatogram spectra. B) Mass spectra.

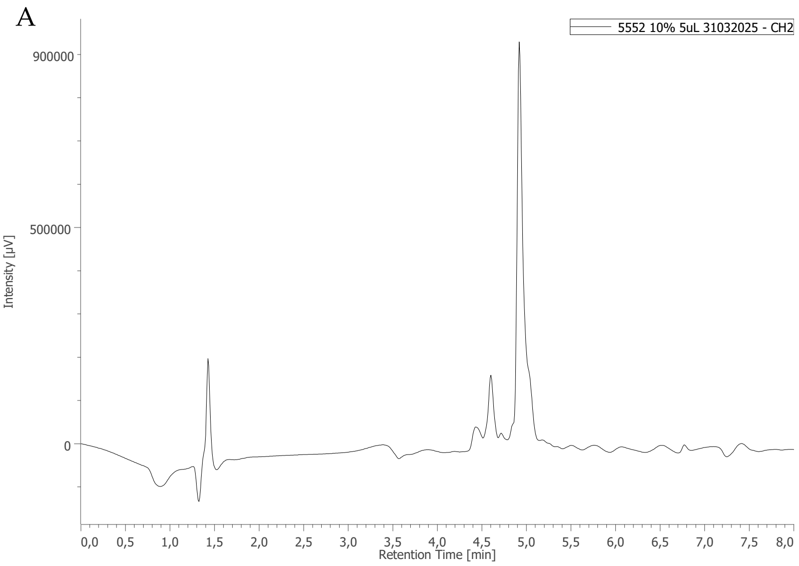

B

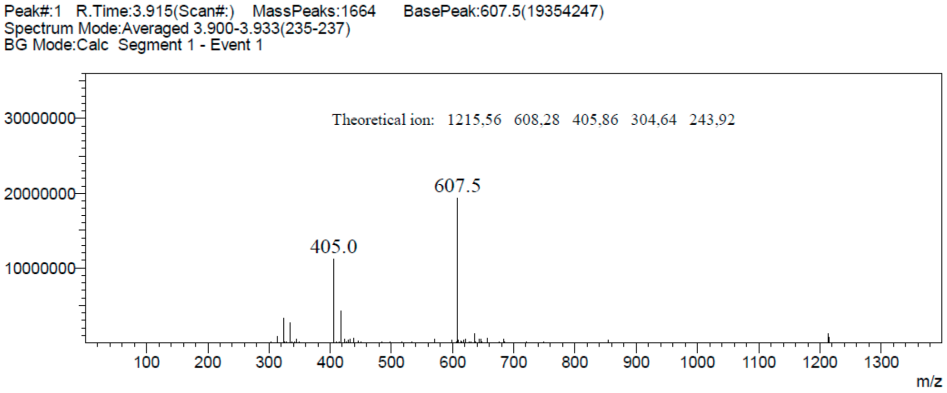

Figure S4. Characterization of R252F-P255M (5553) peptide. A) Chromatogram spectra. B) Mass spectra.

A

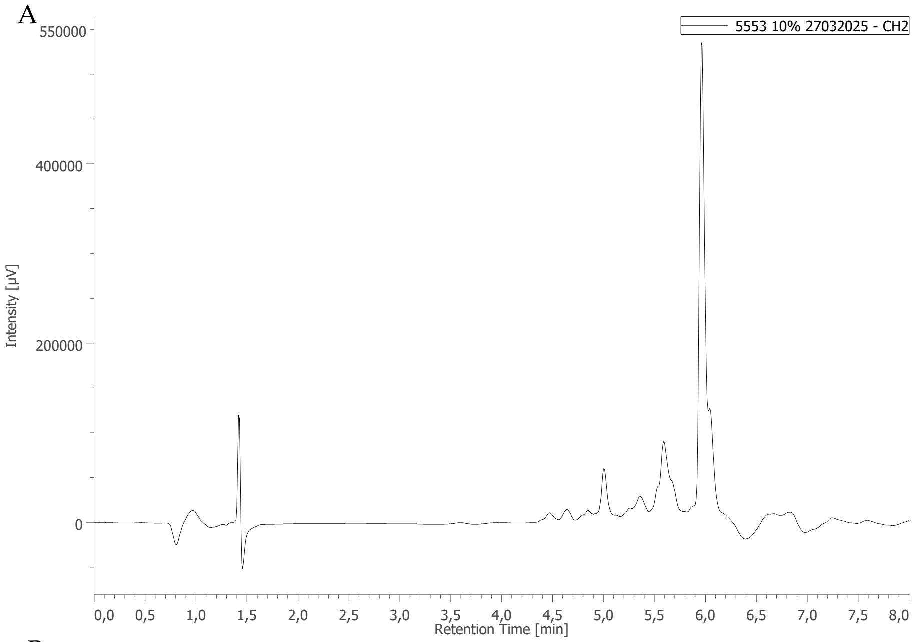

B

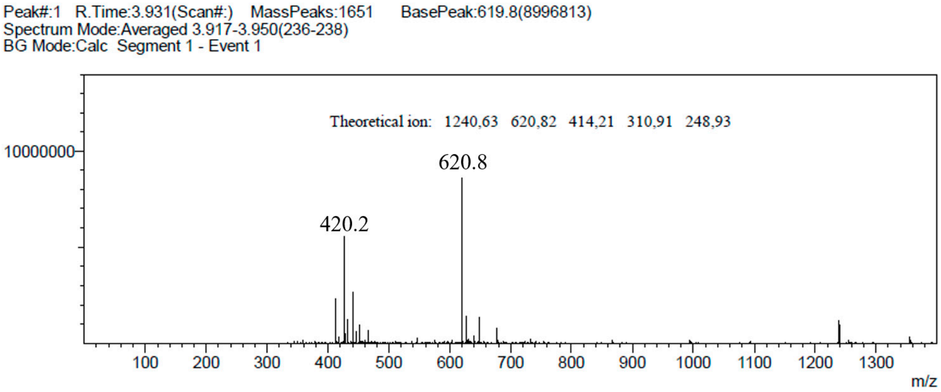

Figure S5. Characterization of Y95-Y107 (5554) peptide. A) Chromatogram spectra. B) Mass spectra.

A

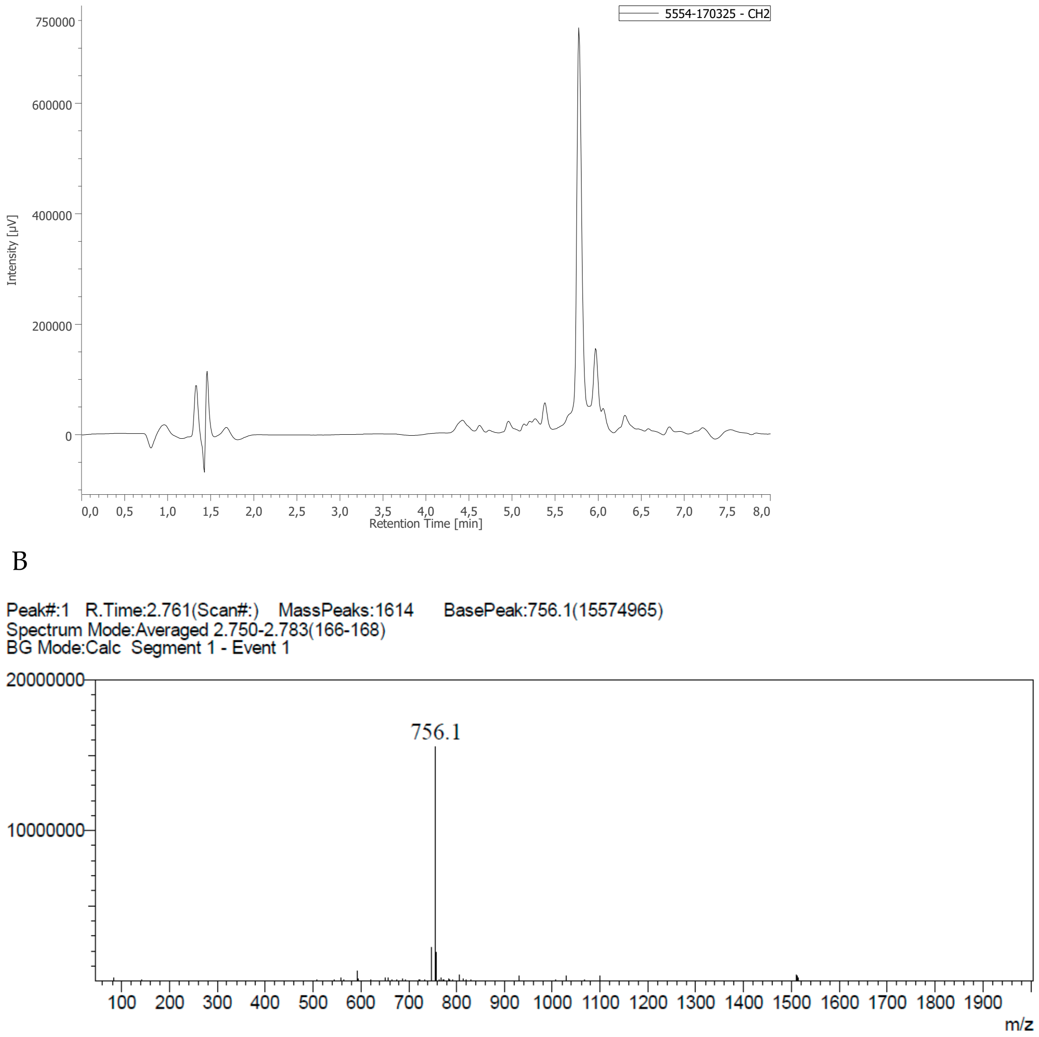

Figure S6. Characterization of N97A-P101L (5555) peptide. A) Chromatogram spectra. B) Mass spectra.

A

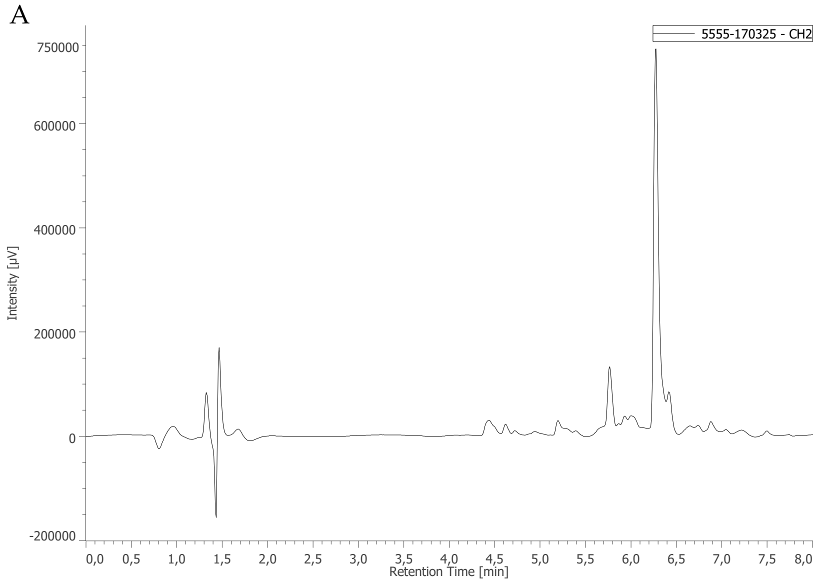

B

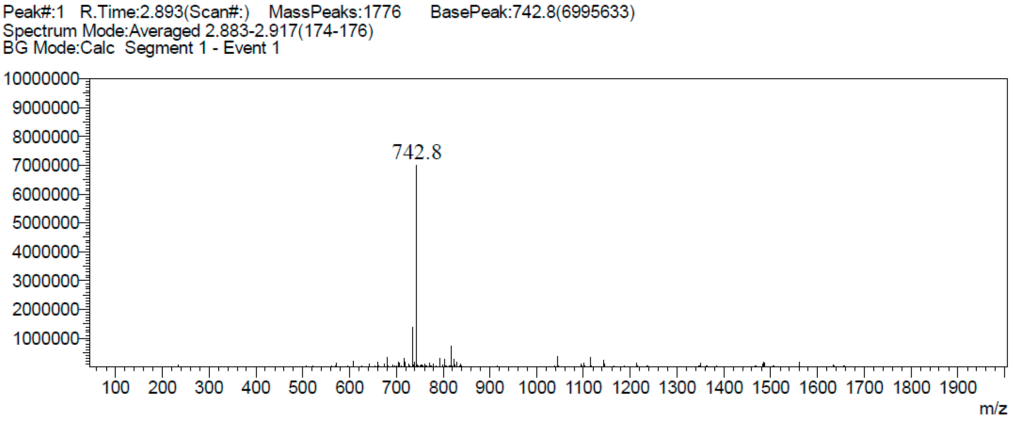

Figure S7. Characterization of N97L-P101L (5556) peptide. A) Chromatogram spectra. B) Mass spectra.

A

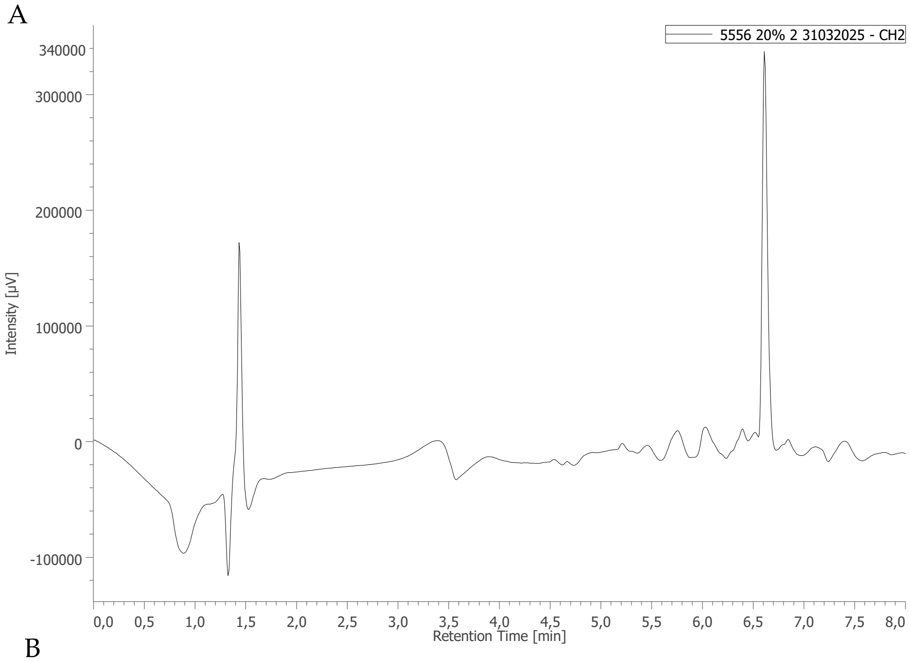

B

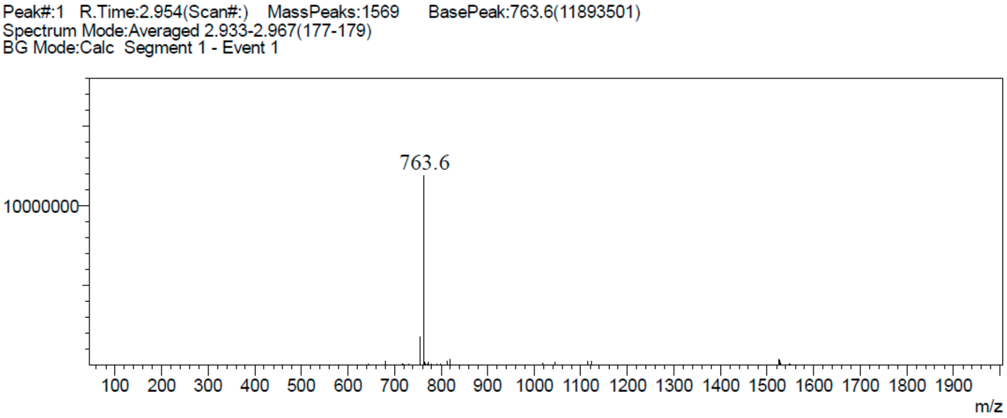

Figure S8. Characterization of Y95-N97D (5557) peptide. A) Chromatogram spectra. B) Mass spectra.

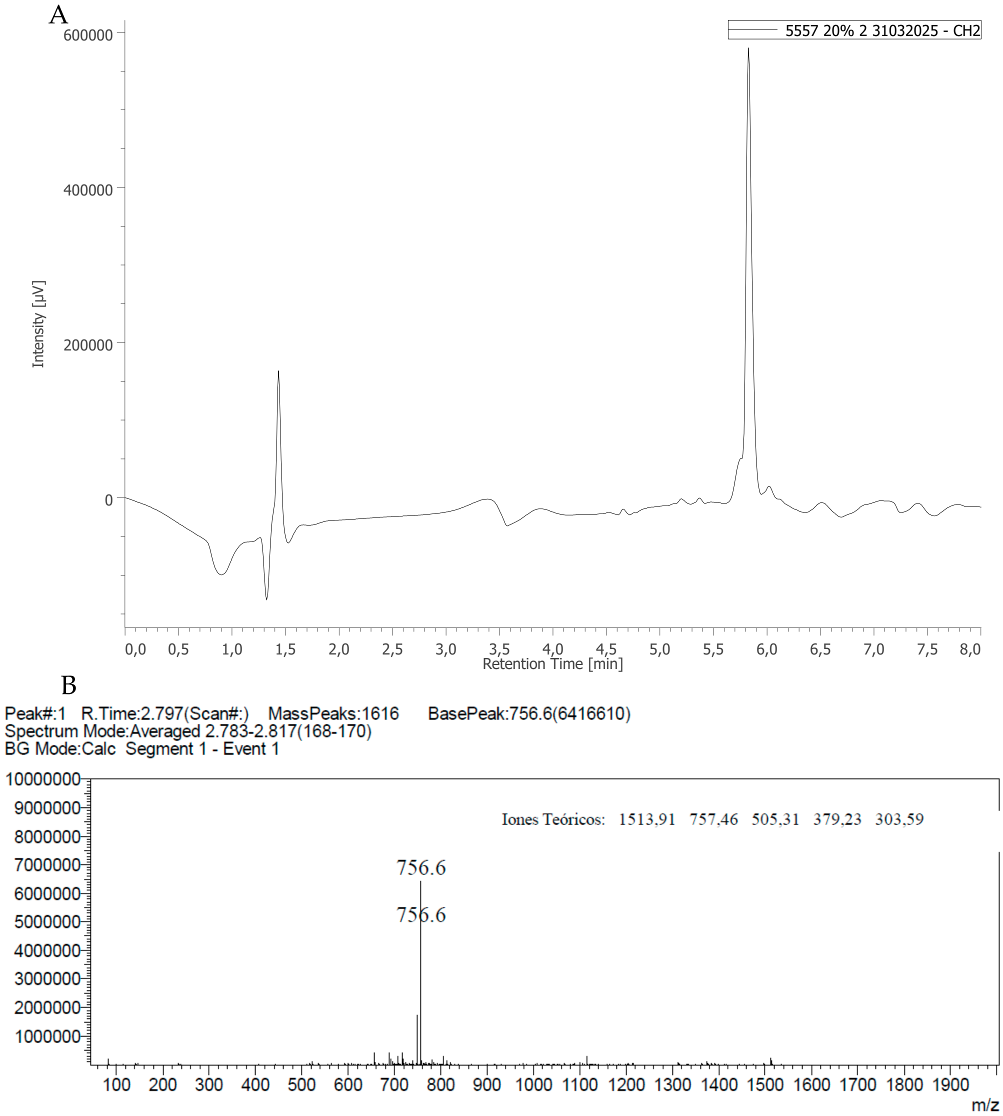

Figure S9. Characterization of P101L-P102H (5558) peptide. A) Chromatogram spectra. B) Mass spectra.

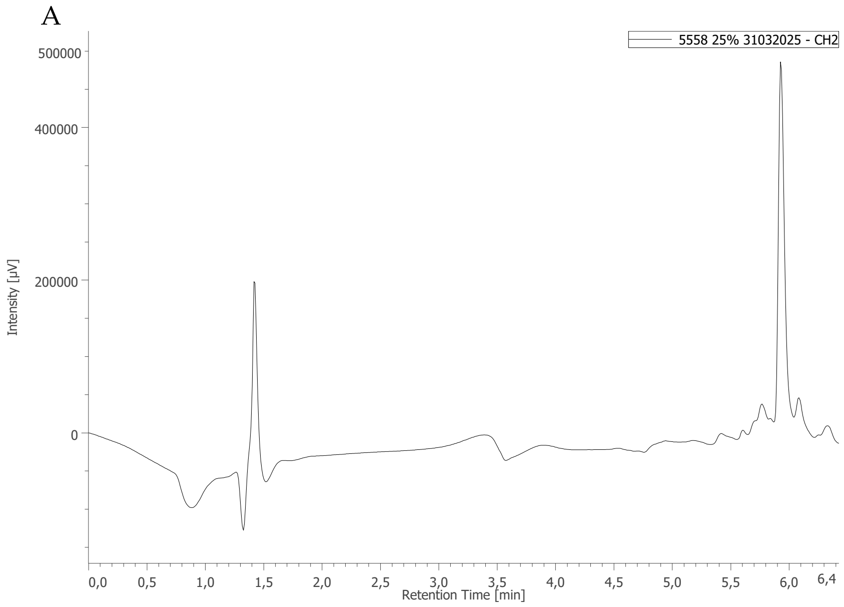

B

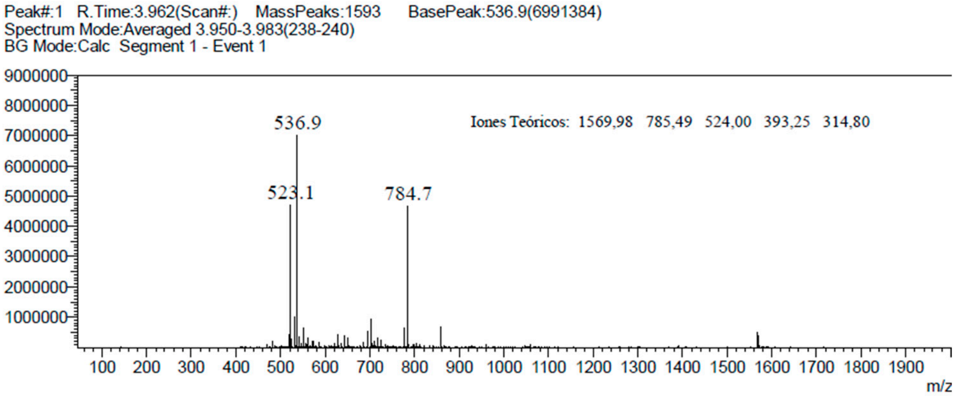

Figure S10. Characterization of P102H-T104L (5559) peptide. A) Chromatogram spectra. B) Mass spectra.

A

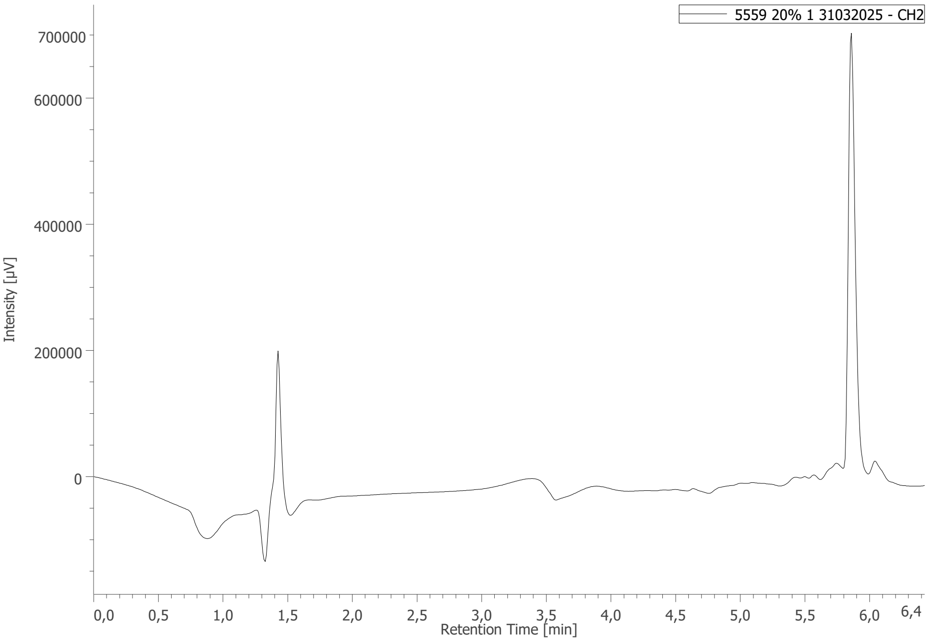

B

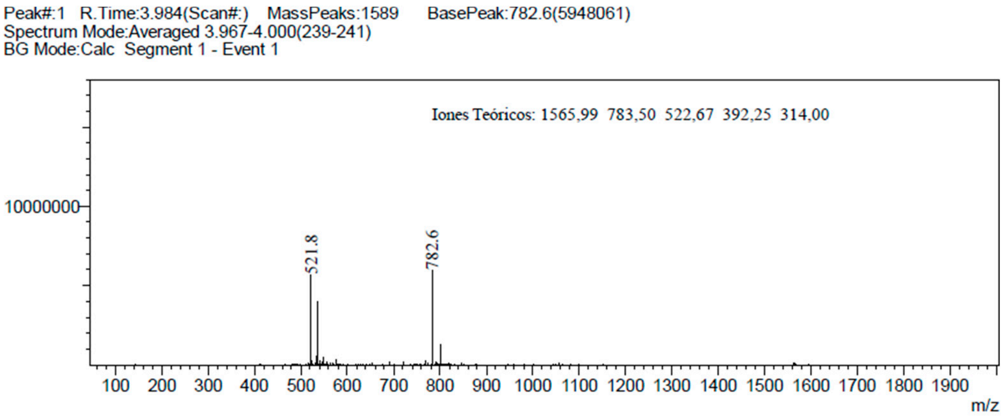

Supplement: Supplementary file 1 [file cancers-18-00451-s001.zip › cancers-3988592-supplementary.pdf]
